# Natural selenium stress influences the changes of antibiotic resistome in seleniferous forest soils

**DOI:** 10.1186/s40793-022-00419-z

**Published:** 2022-05-15

**Authors:** Fang-Fang Wang, Guo-Ping Liu, Fan Zhang, Zong-Ming Li, Xiao-Lin Yang, Chao-Dong Yang, Jian-Lin Shen, Ji-Zheng He, B. Larry Li, Jian-Guo Zeng

**Affiliations:** 1 College of Animal Science, Yangtze University, Jingzhou, 434025 China; 2 State Key Laboratory of Urban and Regional Ecology, Research Center for Eco-Environmental Sciences, Chinese Academy of Sciences, Beijing, 100085 China; 3 University of the Chinese Academy of Sciences, Beijing, 100049 China; 4 College of Horticulture and Gardening, Yangtze University, Jingzhou, 434025 Hubei China; 5 Key Laboratory of Agro-Ecological Processes in the Subtropical Region, Institute of Subtropical Agriculture, Chinese Academy of Sciences, Changsha, 410125 China; 6 Faculty of Veterinary and Agricultural Sciences, The University of Melbourne, Parkville, VIC 3010 Australia; 7 Department of Botany and Plant Sciences, University of California, Riverside, CA 92521-0124 USA; 8 College of Veterinary Medicine, Hunan Agricultural University, Changsha, 410128 China

**Keywords:** Antibiotic resistance genes, Selenium, Soil environment, Bacterial community, High-throughput quantitative PCR, Whole genome analysis

## Abstract

**Background:**

Metal(loid)s can promote the spread and enrichment of antibiotic resistance genes (ARGs) in the environment through a co-selection effect. However, it remains unclear whether exposure of microorganisms to varying concentrations of selenium (Se), an essential but potentially deleterious metal(loid) to living organisms, can influence the migration and distribution of ARGs in forest soils.

**Results:**

Precisely 235 ARGs conferring resistance to seven classes of antibiotics were detected along a Se gradient (0.06–20.65 mg kg^−1^) across 24 forest soils. (flor)/(chlor)/(am)phenicol resistance genes were the most abundant in all samples. The total abundance of ARGs first increased and then decreased with an elevated available Se content threshold of 0.034 mg kg^−1^ (*P* = 2E−05). A structural equation model revealed that the dominant mechanism through which Se indirectly influences the vertical migration of ARGs is by regulating the abundance of the bacterial community. In addition, the methylation of Se (mediated by *tehB*) and the repairing of DNA damages (mediated by *ruvB* and *recG*) were the dominant mechanisms involved in Se resistance in the forest soils. The co-occurrence network analysis revealed a significant correlated cluster between Se-resistance genes, MGEs and ARGs, suggesting the co-transfer potential. *Lelliottia amnigena* YTB01 isolated from the soil was able to tolerate 50 μg mL^−1^ ampicillin and 1000 mg kg^−1^ sodium selenite, and harbored both Se resistant genes and ARGs in the genome.

**Conclusions:**

Our study demonstrated that the spread and enrichment of ARGs are enhanced under moderate Se pressure but inhibited under severe Se pressure in the forest soil (threshold at 0.034 mg kg^−1^ available Se content). The data generated in this pilot study points to the potential health risk associated with Se contamination and its associated influence on ARGs distribution in soil.

**Supplementary Information:**

The online version contains supplementary material available at 10.1186/s40793-022-00419-z.

## Introduction

Antibiotic resistance and/or multidrug resistance of clinical pathogens has become a serious global health problem, which has been aggravated by the increasing overuse of antibiotics [1–3]. Overuse of antibiotics poses selective pressure on bacterial communities and facilitates the dissemination of antibiotic resistance genes (ARGs) in the environment [4]. Indeed, ARGs can be horizontally transferred (commonly termed horizontal gene transfer, HGT) between pathogenic and non-pathogenic bacteria through mobile genetic elements (MGEs) [5]. Metal(loid)s, widely distributed and persistent contaminants of the soil, differ from antibiotics but can serve as a co-selective pressure for antibiotic-resistant bacteria and ARGs [6]. Indeed, several studies have demonstrated that under favorable conditions, metal(loid)s can enhance antibiotic resistance in various soil environments, including multi-metal(loid)s contaminated swine farms soil [7], copper contaminated soil [8], arsenic enriched pig manure [9], as well as arsenic and cadmium polluted paddy soil [10].

Selenium (Se) is an essential nutrient element for many microorganisms and animals, including humans [11]. It exists as different species in the soil, including highly soluble Se oxyanions (selenate and selenite), less mobile elemental Se and reduced selenides [12]. In organisms that need Se, selenocysteine is involved in the formation of selenoproteins and plays an important role in detoxifying and scavenging reactive oxygen species (ROS) [13, 14]. Also, a moderate concentration of Se is beneficial to the growth of bacteria, while a high concentration of Se causes deleterious effects [15]. When bacteria were exposed to toxic concentrations of selenite, intracellular selenite acted as a pro-oxidant, leading to the accumulation of ROS, which in turn caused damage to proteins and DNA [16, 17]. To resist the toxicity of selenite, a series of Se resistance genes, including superoxide dismutase (*sodA* and *sodB*) [18], DNA damage repair enzymes (*recG* and *ruvB*) [19], and tellurite-resistance genes (*tehB*) [20] coordinate the detoxification and repair process. Experimental evidence has shown that Se-enriched bacteria have a stronger ability to scavenge ROS and are more resistant to antibiotics than non-Se-enriched bacteria [21]. In addition, analyses of massive complete genome collections found close genetic linkages and high co-transfer potentials between Se resistance genes and bacitracin as well as between kasugamycin and β-lactam resistance genes [22]. Therefore, we hypothesize that Se pressure may affect the distribution of ARGs in soils.

In humans, Se has many important biological functions, including anti-oxidation and anti-cancer activity [23]. Studies have shown that some populations living in soil Se deficient areas, including China, Europe and New Zealand, lack adequate Se intake and, therefore, are at risk of Se deficiency [24]. Developing Se-enrich agricultural foods have been recognized as an effective strategy to argument the Se intake of humans [25, 26]. The application of selenite fertilizers in agriculture or the addition of selenite feeds in animal husbandry is gaining popularity [27]. However, overuse of selenite fertilizers may contaminate the soil. Since metal(loid)s have been found to drive the spread of ARGs in soil ecosystems [7–10], it remains unclear whether the exposure of microorganisms to Se pressure may promote the enrichment and migration of ARGs in soils.

Forests are an important component of terrestrial ecosystems globally and a huge ARGs reservoir for exchange with clinical pathogens [28]. The Enshi Autonomous Prefecture of Hubei province, China, is a natural Se-rich area where the average soil Se content reaches 9.6 mg kg^−1^ (the world average Se content is 0.2 mg kg^−1^) [29]. The Yutangba area, located in the north of Enshi Autonomous Prefecture, is a typical high-Se basin, with soil Se content ranging from 0.41 to 42.3 mg kg^−1^ [29]. Seleniferous forest soils with low anthropogenic influences are ideal areas to investigate the relationship between Se exposure and the distribution of ARGs as well as the underlying mechanisms. Therefore, we aimed to investigate the patterns of ARGs and bacterial community along a Se gradient by analyzing soils from 24 forest sites in Enshi. Furthermore, we used a culture-dependent approach to investigate the association between Se-enrichment and antibiotic resistance in bacteria. Lastly, we investigated the genetic mechanisms of the association between Se-enrichment and antibiotic resistance by analyzing the whole genome of Se-enriched bacteria.

## Methods

### Study area and soil sampling

**Fig. 1:**
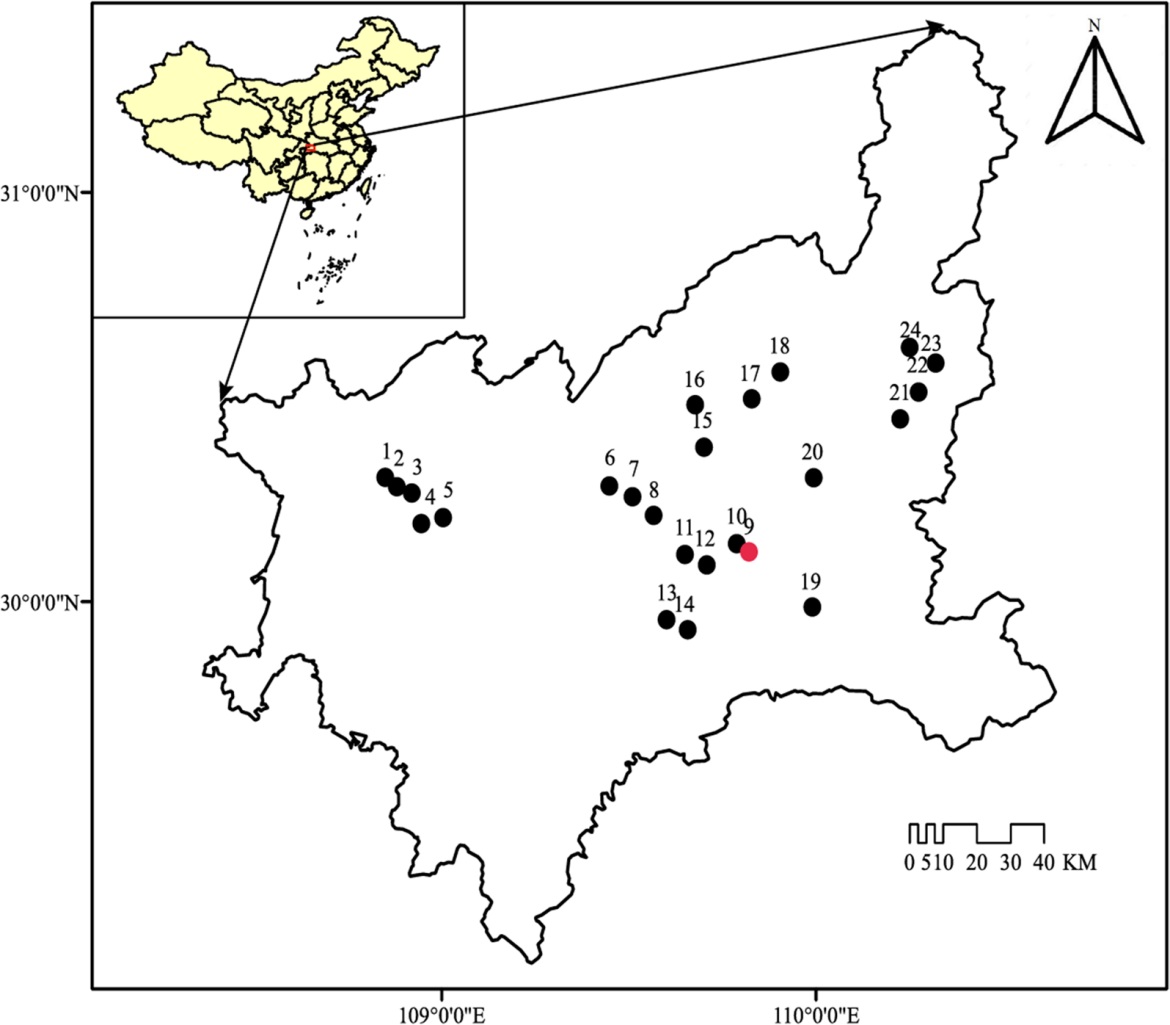
Map of the study area. Twenty-four sampling sites around the high Se content area (red point) in the forest of Enshi Autonomous Prefecture of Hubei province, China, were sampled

Soil samples were collected in the forest of Enshi, an autonomous prefecture located in the southwest mountain area of Hubei Province, China (see Fig. 1 and Additional file 7: Table S1 for geographic information on the area). The sampling area is a subtropical monsoon climate with an annual average temperature of 16.2 °C and annual average precipitation of 1600 mm [30]. Based on previous studies which fully investigated the soil Se content in our sampling region, we selected 24 sampling sites around the Se-rich Yutangba area (Fig. 1). To the best of our knowledge, animal manure or fertilizers have never been applied to the soils in these sampling sites. Also, prior to sampling, these sites have experienced very minimal human impacts. In each site, three replicate samples were obtained randomly from a 20 m × 20 m plot. After removing the litter layer, samples were aseptically collected from the topsoil (10 cm depth) using a sterile spade. Three soil sub-samples were collected from 1 m × 1 m sub-plot and mixed to form a composite sample. Composite samples were immediately placed on ice and transported to the laboratory within 24 h of collection. Soil sub-samples for the isolation of culturable bacteria were stored at 4 °C, and soil sub-samples for the extraction of total microbial community DNA were freeze-dried and stored at − 80 °C. The soil sub-samples for the analyses of chemical properties were air-dried, crumbled to pass through a 2-mm sieve and thoroughly homogenized using standard methods.

### Chemical properties analyses and Se determination

The soil water content was determined gravimetrically by drying the soil samples at 105 °C for 24 h. Soil pH was determined with a soil to water ratio of 1: 2.5 using a pre-calibrated FiveEasy Plus™ pH-meter (Mettler Toledo, Zurich, Switzerland). The total soil carbon (TSC) and total soil nitrogen (TSN) were determined on an automated VarioMax CN Element Analyzer (Elementar, Hanau, Germany). Soil total phosphorus was measured with Mo-Sb colorimetric method using a spectrophotometer (Shimadzu, Kyoto, Japan). Soil total potassium was digested with hydrofluoric acid and perchloric acid and subsequently measured using a flame photometer (Shanghai Jing Ke Instrument Co., Ltd, Shanghai, China).

Se contents in soil were determined using HG-AFS (coupling of hydride generation to atomic fluorescence spectrometer), a sensitive method for the determination of Se [31]. Preprocessing of samples and determination of total and available Se contents were conducted according to the method of García et al. [31] with slight modifications (Additional file 8: Text S1). Se content in the samples was determined by extrapolation to a standard curve of Se concentrations analyzed using a double-channel atomic fluorescence spectrometry (AFS-930, Beijing Titan Instruments Co., Ltd, Beijing, China). The recovery rate of soil quality standard GBW07428(GSS-14) was 90–110%.

### Antibiotic residue quantitation

Two target tetracyclines, namely tetracycline (TCN) and doxycycline hydrochloride (DOX); two (fluoro)quinolones, namely ofloxacin (OLF) and enrofloxacin (ENR); two *β*-lactam, namely amoxicillin (AMX) and clavulanic acid (CA); the aminoglycoside kanamycin (KAN); and the erythromycin (ENY), a macrolide lincosamide streptogramin_b (MLSB), were quantified in this study. The antibiotic residues in soils were extracted using solid-phase extraction [32]. Thereafter, the concentrations of antibiotics were determined using liquid chromatography-tandem mass spectrometry (LC-MS/MS) as previously described [33].

### Determination of the profile and abundances of ARGs and MGEs through high-throughput quantitative PCR (HT-qPCR)

High-throughput quantitative PCR (HT-qPCR) was used to determine the profile and abundance of ARGs and MGEs in soil samples. The profile of MGEs was targeted in order to measure the potential of horizontal gene transfer of ARGs. Firstly, microbial DNA was extracted from 0.25 g soil sample with the MoBio PowerSoil DNA isolation kit (Qiagen, Hilden, Germany) following the manufacturer's instruction. The quantity and quality of the extracted DNA were determined using a NanoDrop ND2000c spectrophotometer (NanoDrop Technologies, Wilmington, DE, USA). The DNA was kept at − 20 °C until analyses.

Next, the profile of ARGs and MGEs were measured using HT-qPCR array on a CFX384™ Real-Time system (Bio-Rad Laboratories, Hercules, CA, USA) [34]. The HT-qPCR array contains 364 pairs of primers which can simultaneously target 328 ARGs including seven main classes of antibiotics (aminoglycosides, β-lactam, erythromycin, (flor)/(chlor)/(am)phenicol, MLSB, tetracycline, and vancomycin), and series of multidrug/efflux genes; 34 MGEs, including transposon-transposase genes, integron-integrase genes and plasmids; and 2 replicate 16S rRNA genes (housekeeping genes). Each 10 μL qPCR reaction contained 5 μL of Premix Ex Taq™ (TaKaRa Biotechnology, Dalian, China), 0.5 μL of each primer (10 mM), 1 μL template DNA, and nuclease-free water. The thermal cycling conditions were: 95 °C for 10 min and then 40 cycles of denaturation at 95 °C for 30 s and annealing at 60 °C for 30 s. Each reaction was conducted in triplicate and contained negative controls. The qPCR amplification efficiency ranged from 80 to 120%. The relative abundance of ARGs and MGEs was determined after dividing target gene abundance by the bacterial 16S rRNA gene copy number [35].

The quantitative PCR of the 16S rRNA gene was conducted on a CFX96™ Real-Time system (Bio-Rad Laboratories) by standard curve method [36]. The primer set was Bact1369F/Prok1492R with the Probe^TM^1389F [37]. The reaction wells contained 12.5 μL Premix Ex Taq™ (Takara Biotechnology, Dalian, China), 10 μM of each primer, 5 ng μL^−1^ template DNA, 1 μL Probe^TM^1389F and nuclease-free water to a final reaction volume of 25 μL. Information on the plasmid DNA used to generate the standard curve and the amplification procedure are described in a previous study [38].

### Absolute quantification of Se resistance genes

Se resistance genes, including *recG*, *ruvB*, *sodA*, *sodB* and *tehB* were retrieved from BacMet (http://bacmet.biomedicine.gu.se/). The genes *recG* and *ruvB* are ATP-dependent DNA helicases involved in repairing DNA damage caused by selenite [19], while *sodA* and *sodB* are Mn- and Fe-dependent superoxide dismutases, respectively [18]. The products of *sodA* and *sodB* are involved in eliminating ROS caused by excessive intracellular selenium. The *tehB gene* is a S-adenosyl-L-methionine-dependent methyltransferase involved in the methylation of Se and responsible for Se detoxification [20]. In addition, based on published research, we targeted eleven Se metabolism genes, including two methyltransferase genes *mmtA* and *ubiE* [39], two selenocysteine synthesis genes *SelA* and *SelB* [40]*,* two groups of selenate reductase: *serA*, *serB, serC and serD* (group 1) [41] and *srdA, srdB, srdC* (group 2) [42]. Libraries for all the above sixteen genes were constructed via PCR cloning and generation of recombinant T-Vectors harboring the 16 genes. Thereafter, these plasmid DNAs were used to generate standard curves and amplification profiles for the absolute quantification of the abundance of the sixteen genes in soil samples on a CFX96™ Real-Time system (Bio-Rad). The qPCR was a 10 µL reaction volume and included 5 µL Premix Ex Taq™ (Takara Biotechnology, Dalian, China), 0.5 µL each primer, 0.5 µL template DNA, and nuclease-free water. The primer sequences and PCR conditions are provided in Additional file 7: Table S2. Each PCR reaction was performed in triplicate and included negative controls. The amplification efficiency was valid (ranged from 80 to 120%). The relative abundance of Se resistant genes was determined after dividing target gene abundance by the bacterial 16S rRNA gene copies.

### Bacterial 16S rRNA gene sequence-based diversity analysis

The bacterial community composition was determined by amplifying the hypervariable V4 region of the bacterial 16S rRNA gene with primer pair 515F and 907R [43]. PCR reactions were performed on GeneAmp 9700 thermal cycler (Applied Biosystems, Waltham, MA, USA) and conducted in triplicate. Each PCR reaction contained 5 μL of Premix Ex Taq™ (TaKaRa Biotechnology, Dalian, China), 0.5 μL of each primer, 10 ng template DNA and nuclease-free water in total 10 μL volume. The thermocycler condition was 94 °C for 3 min, 27 cycles of 95 °C for 30 s, 55 °C for 30 s and 72 °C for 45 s at, with a final extension at 72 °C for 10 min. The PCR products were purified using the AxyPrep DNA Gel Extraction Kit (Axygen Biosciences, Union City, CA, USA). The sequencing was performed on an Illumina MiSeq system (Illumina, San Diego, CA, USA) at Majorbio Bio-Pharm Technology, Shanghai, China.

Raw sequence reads were processed using the Quantitative Insights Into Microbial Ecology (QIIME, version 1.9) with the suggested operational procedure [44, 45]. Usearch *v10.0* [46] was used to merge quality-filtered forward and reverse reads as well as eliminate chimeric sequences. Thereafter, the sequence reads were clustered into operational taxonomic units (OTUs) at 97% 16S rRNA gene similarity, and representative sequences aligned against the Silva reference database (Release 132) for taxonomic assignment (https://www.arb-silva.de/). All 16S rRNA gene sequences have been uploaded to Bio-Med Big Data Center (https://www.biosino.org/node/index) with the accession number OEP001738.

### Isolation of Se-resistant bacteria and growth kinetics

To investigate the association between a high concentration of Se and resistance genes in bacteria, we selected two soil samples that were respectively collected from sites with the highest and lowest soil selenium contents. The soil samples were serially diluted (up to 10^−3^) with normal saline (0.85% NaCl) and inoculated onto tryptic soy broth (TSB) (1:200), supplemented with 50 μg mL^−1^ ampicillin and 0, 1, 10, 100 or 1000 mg kg^−1^ sodium selenite. The ampicillin concentration and the gradient of sodium selenite concentration were determined according to preliminary experiments. That is, the concentrations of 50 μg mL^−1^ ampicillin and 1000 mg kg^−1^ sodium selenite inhibited the growth of most sensitive bacteria as determined by enumeration of colony forming unit. Each ampicillin-sodium selenite combination was set up in triplicates. Cultures were incubated at 28 °C for 48 h with gently shaking at 50 rpm. Bacterial growth was monitored every 6 h by optical density measurements at a wavelength of 600 nm. Subsequently, for enumeration of viable bacteria, cultures in the middle and early stages of the logarithmic growth phase were selected and spread plated on tryptic soy agar (TSA) plates supplemented with 3000 mg kg^−1^ sodium selenite. The cultures were then inoculated in TSB supplemented with a series of sodium selenite (0, 1, 10, 100 or 1,000 mg kg^−1^) and 50 μg mL^−1^ ampicillin to generate a growth curve. Furthermore, to isolate Se-resistant bacteria, three single clones were picked up from each of the above TSA plates and then cultured in TSB. Finally, sodium selenite susceptibility assays were performed on the pure cultures by growing them in TSB amended with 50 μg mL^−1^ ampicillin and 0, 500, 1000, 3000, 6000, or 9000 mg kg^−1^ sodium selenite at 28 °C with shaking for 48 h. Thereafter, the optical density was determined at 600 nm with a spectrophotometer.

### Genome sequencing, assembly and gene annotation

The DNA of the pure microbial cultures was extracted using the QIAamp DNA Microbiome Kit (Qiagen, Hilden, Germany) by following the manufacturer’s instructions. Thereafter, the V4 region of the 16S rRNA gene was sequenced using Sanger sequencing [43]. The complete genome sequencing of the isolates was performed on the PacBio RS II platform (Pacific Biosciences of California, Inc, Menlo Park, CA, USA) and Illumina HiSeq 4000 platform (Illumina Inc., San Diego, CA, USA) at the Beijing Genomics Institute (BGI, Shenzhen, China).

The sequences of the V4 region of the 16S rRNA gene were manually checked for quality and edited using Chromas software, and aligned against the NCBI GenBank using BLAST to identify the bacterial species. The complete genome sequence data was assembled using multiple software, including Falcon *v0.3.0*, proovread *v2.12* and Celera Assembler *v8.3* as previously reported [47–49]. The genome gene prediction was performed using glimmer3 (http://www.cbcb.umd.edu/software/glimmer/) [50, 51]. The genome gene was annotated through the NCBI PGAP using BLAST (https://blast.ncbi.nlm.nih.gov/Blast.cgi). Seven databases, including Kyoto Encyclopedia of Genes and Genomes (KEGG, https://www.genome.jp/kegg/), Clusters of Orthologous Groups (COG, https://www.ncbi.nlm.nih.gov/research/cog-project/), Non-Redundant Protein Database (NR) [52], Swiss-Prot [53], TrEMBL [53], Gene Ontology (GO) [54], and EggNOG *v5.0* [55] were used for general functional annotation. Virulence factors and drug resistance genes were identified using the Virulence Factors of Pathogenic Bacteria database (VFDB, http://www.mgc.ac.cn/VFs/main.htm) and The Comprehensive Antibiotic Resistance Genes Database (CARD, https://card.mcmaster.ca/) [56], respectively.

To obtain insights into whether co-regulation between Se metabolism and antibiotic resistance exists, the whole genome sequence information was further mined for the presence of secondary metabolite biosynthetic gene clusters (BGCs) using antiSMASH *v6.0* [57]. The complete genome sequences are available in the Bio-Med Big Data Center (https://www.biosino.org/node/index) under the accession number OEP001741.

### Statistical analysis

Pearson correlation and normality test were performed in SPSS 21 (IBM, Armonk, NY, USA). Five models, namely linear, quadratic, cubic, segmented, and stegmented regression, were used to fit the relationship between available Se content and the detected number of ARGs or relative abundance of ARGs with the “chngpt” package in R (*v.3.4.1*) (https://www.r-project.org/) [58]. Redundancy analysis (RDA) was performed to investigate the relationship of soil chemical properties, bacterial phylum, relative abundance of MGEs and Se resistance genes on the pattern of ARGs using the “vegan” package of R [59]. Structural equation modeling (SEM) was used to examine the direct and indirect effect of edaphic factors, Se resistance genes, bacterial abundance and MGEs on the pattern of ARGs. SEM was constructed and analyzed using the “lavaan” [60] and “piecewiseSEM” packages [61] of R, respectively. For the SEM, the first principal coordinate of the relative abundance matrix of Se resistant genes, MGEs, ARGs and phylum-level abundance of bacterial community were extracted with the “vegan” package of R. The goodness of fit was tested by “fitMeasures” function in “lavaan” package. Co-occurrence network analysis was performed to explore the interactive correlation among the relative abundance of ARGs, MGEs, and Se resistance genes (*r* > 0.8, *P* < 0.05). The pairwise correlations were calculated by Pearson correlation analysis using the "psych" package [62] in R and then visualized and further annotated using Gephi *v0.9.2* [63].

## Results

### Se contents and major soil characteristics

Among all samples, soil total Se content ranged from 0.06 to 20.65 mg kg^−1^_,_ with a mean value of 3.40 mg kg^−1^ (Additional file 7: Table S1), which was higher than the world mean total Se value of 0.2 mg kg^−1^ [29]. The highest total Se content sample, with a mean content of 20.65 mg kg^−1^, was collected from TZY2 site. Soil available Se content ranged from 0.003 to 0.14 mg kg^−1^ with a mean of 0.03 mg kg^−1^. Pearson correlation analysis showed that pH and available Se content were significantly positively associated (*r* = 0.61, *P* < 0.05) (Additional file 7: Table S3). In addition, available Se and total Se contents were significantly positively correlated (*r* = 0.90, *P* < 0.05). Given that available Se is the bioavailable form of Se in the soil [12], it was selected for further analyses instead of total Se content.

### Diversity and abundance of ARGs and MGEs

**Fig. 2:**
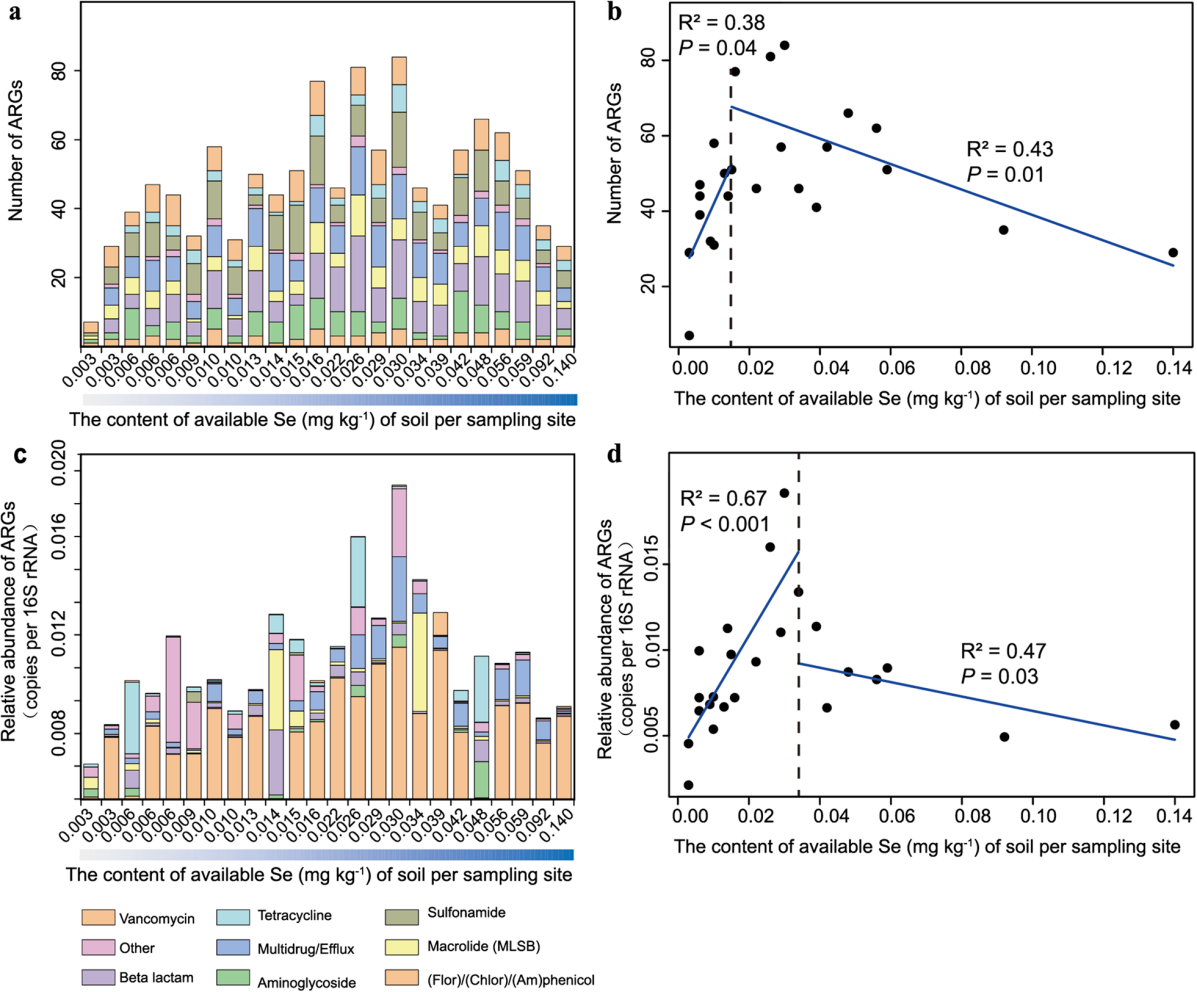
ARGs abundance and correlation with Se content. **a** The number of detected ARGs. **b** The relative abundance of ARGs. **c** Correlation between available Se content and the detected number of ARGs. **d** Correlation between available Se content and the relative abundance of ARGs (**d**). In **a**, **c**, ARGs conferring resistance to seven classes of antibiotics or multidrug/efflux were shown in columns of different colors. Others refer to ARGs are not included in the seven classes of antibiotics. Blue solid lines are segmented according to the threshold regression model and show the significant relationship below and above the threshold (**b**, **d**)

A total of 264 genes, including 235 ARGs and 29 MGEs, were detected in all samples (Additional file 1: Fig. S1). The numbers of detected ARGs in each sample ranged from 7 to 84 (Fig. 2a). The detected ARGs confer resistance to seven classes of antibiotics, including aminoglycoside, β-lactam, (flor)/(chlor)/(am)phenicol, sulfonamide, tetracycline, MLSB, and vancomycin, and also codes for a series of multidrug/efflux proteins. The detected number of ARGs coding for multidrug/efflux pumps was the highest, accounting for 18.9% of the total numbers of ARGs. ARGs for β-lactam (17.8%) and tetracycline (17.2%) were the next most abundant after multidrug/efflux pumps. The relative abundance of ARGs detected in these seleniferous soil samples ranged from 2.30E−06 to 9.25E−03 copies per 16S rRNA gene copy (Fig. 2b). Among them, multidrug resistance genes were detected in the highest abundance (63.2% of total ARGs) in all samples. Among the 235 ARGs, 11 highly abundant genes were detected in as much as 79.2% (19/24) of samples, including five multidrug/efflux genes (*oprJ*, *mexF*, *acrR-03*, *acrA-04*, *acrA-05*), two β-lactam resistance genes (*oprD*, *fox5*), one MLSB resistant gene (*oleC*), one aminoglycoside resistant gene (*aadA1*), one tetracycline (*tetPB-01*), and one other type of ARGs (*sat4*). *MexF*, an important multidrug efflux gene, had the highest abundance with a mean relative abundance of 5E−03 copies per 16S rRNA copy across all samples. The normalized copy numbers of MGEs ranged from 9.79E−06 to 1.32E−01 copies per bacterial cell. The *intI1* gene encoding class 1 integron-integrase was detected in only 3 of the 24 samples, with a mean of 5.52E−05 copies per bacterial cell. In addition, Pearson correlation analysis showed that the total abundance of MGEs was not correlated with the total abundance of ARGs in each sample (data not shown).

### Concentration of antibiotics residues

To investigate whether the ARGs pattern was affected by antibiotics residues in soil, the concentrations of eight major classes of antibiotics residues were analyzed. As shown in Additional file 7: Table S4, the concentrations of amoxicillin (β-lactam class) and tetracycline (tetracycline class) were between 0 and 12.42 μg kg^−1^ and between 0 and 0.45 μg kg^−1^, respectively. The concentrations of the other six antibiotics were below the detection limit in all samples. Furthermore, the Pearson correlation analysis showed that the relationship between the concentration of detected antibiotics and the diversity or abundance of ARGs was not significant (*P* > 0.05).

### 16S rRNA gene quantification and bacterial diversity

**Fig. 3:**
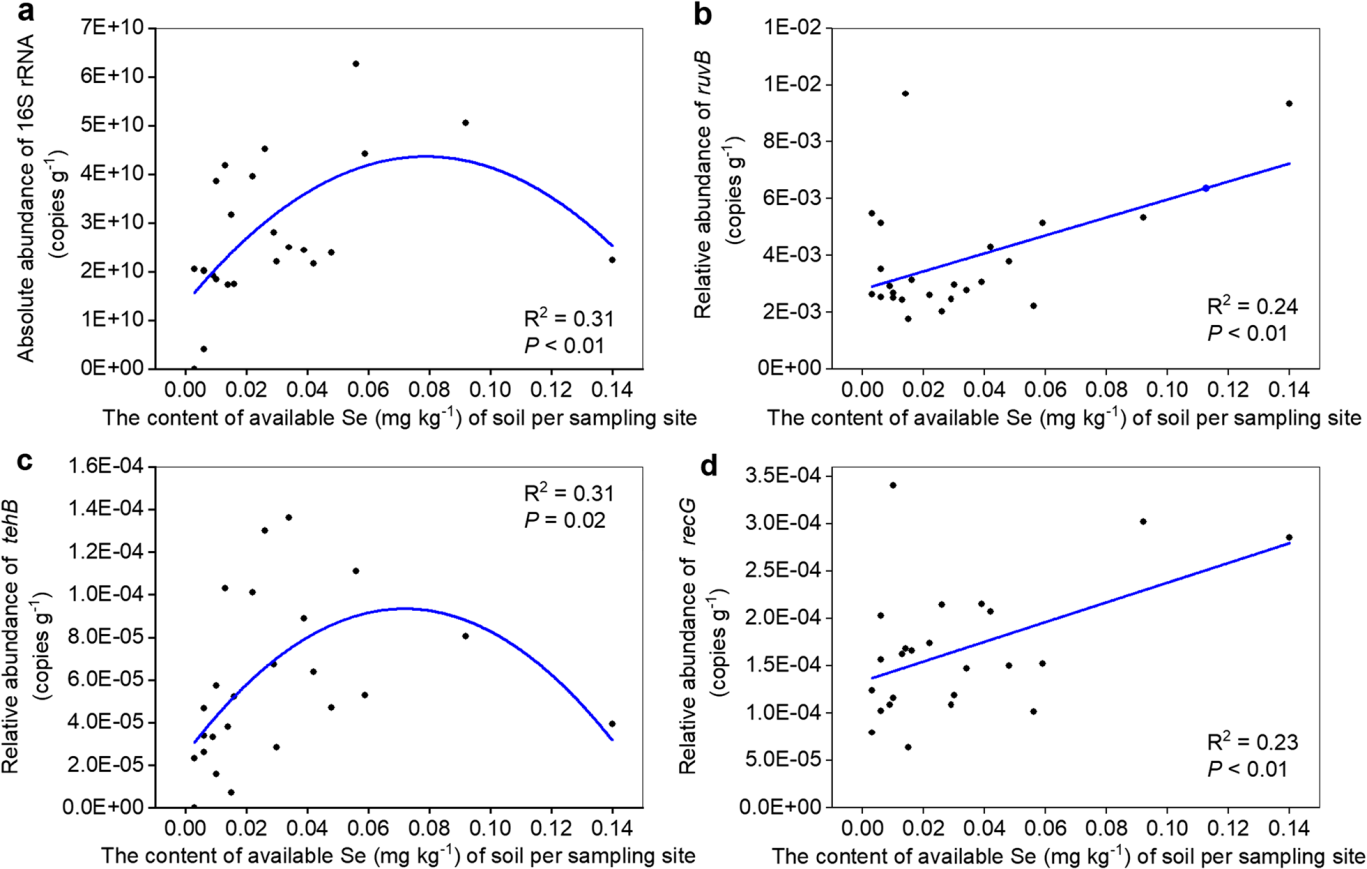
The correlation between available Se content and the absolute copy number of genes. **a** 16S rRNA. **b** *ruvB.* **c** *tehB*. **d** *recG*. Significant quadratic correlations were well fitted between available Se content and the abundance of 16S rRNA and *tehB*. Significant positive correlations were found between available Se content and the abundance of *ruvB* and *recG*

The absolute copy number of 16S rRNA genes (for the total abundance of the bacterial community) ranged from 4.00E+09 to 6.26E+10 copies g^−1^ soil. Based on a second-order polynomial fit, we found that the absolute copy number of the 16S rRNA genes exhibited a unimodal relationship with available Se content (R^2^ = 0.312, *P* < 0.001) (Fig. 3a) and peaked at 0.08 mg kg^−1^ available Se content.

From high-throughput amplicon sequence analysis of the V4 hypervariable region of the 16S rRNA gene, a total of 1,486,320 high-quality sequences (50,006 to 71,070 per sample) were obtained and clustered into 1,178,016 OTUs (38,072 to 58,547 for each sample; mean = 49,533). Proteobacteria, Acidobacteria, Actinobacteria and Planctomycetes were the dominant phyla in all the samples, accounting for 11.76% to 33.39% of total bacterial 16S rRNA gene sequences (Additional file 2: Fig. S2). At the class taxonomic level, Acidobacteria, Alphaproteobacteria, and Actinobacteria were the dominant bacteria, accounting for 22.94%, 17.79%, 12.58% of the total 16S rRNA gene sequences, respectively.

### Quantitative analysis of sixteen Se resistance genes

To investigate how Se resistance genes impact Se distribution and ARGs transfer in these forest soil under Se selective pressure, we constructed libraries of sixteen Se resistance genes and then performed absolute quantitative PCR to determine their abundance in soils across a Se content gradient. Amplifications were obtained for only 12 of the 16 genes, and these were further analyzed. The absolute copy number of Se resistance genes varied from 1.33E+02 copies g^−1^ soil to 5.40E+08 copies g^−1^ soil (Additional file 7: Table S5). Of the 12 genes, *ruvB* was the most abundant, with a mean copy number of 1.93E+08 per gram of soil. Interestingly, the relative abundance of *ruvB*, *tehB,* and *recG* were significantly correlated with available Se content (Fig. 3b–d). Specifically, the relative abundance of *tehB* exhibited a significant quadratic relationship with available Se content (R^2^ = 0.31, *P* = 0.02) while the copy numbers of *ruvB* and *recG* exhibited a positive correlation with available Se content (*ruvB*: R^2^ = 0.24, *P* < 0.01; *recG*: R^2^ = 0.23, *P* < 0.01). More specifically, a threshold for available Se content existed at 0.08 mg kg^−1^, at which point, the relative abundance of *tehB* exhibited a unimodal relationship. The abundance of *tehB* first increased with elevated available Se ranging from 0.003 to 0.008 mg kg^−1^ and then decreased with available Se ranging from 0.08 to 0.14 mg kg^−1^.

### Network analysis

**Fig. 4:**
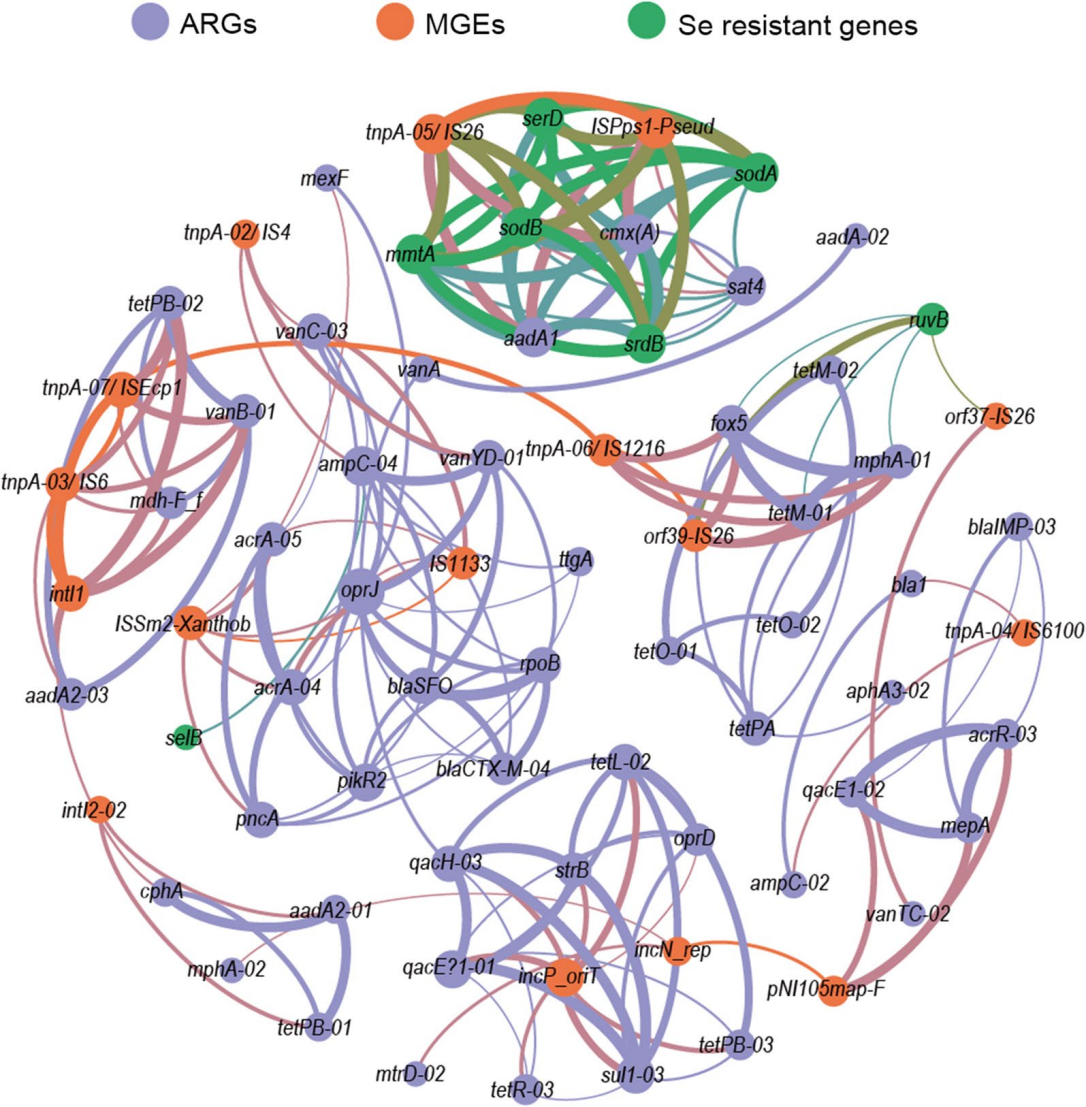
Co-occurrence network analysis showing the correlation between ARGs, MGEs, and Se resistance genes (Spearman' r > 0.8, *P* < 0.05). Node size indicates connectivity, and the color of nodes is grouped by resistance type

The co-occurrence pattern between ARGs, Se resistance genes, and MGEs was explored by network analysis (Fig. 4 and Additional file 3: Fig. S3). The modularity index of the co-occurrence network is 0.797, indicating a high modularity of the network. The network contained six modules, with tightly connected nodes within each module and few cross-connections between different modules. Module I exhibited a close and independent cluster, which consisted of five Se resistance genes, three ARGs, one transposon, and one resistant plasmid. The other five modules were composed of ARGs, Se resistance genes, or MGEs (Fig. 4 and Additional file 3: Fig. S3).

### Correlation between environmental factors, Se resistance genes, MGEs, bacterial community and ARGs

The minimum Akaike information criterion (AIC) value showed that the stegmented regression model was the best fit, and that the thresholds for available Se content were 0.015 mg kg^−1^ (available Se versus the detected number of ARGs, *P* = 8E−04) (Fig. 2c) and 0.034 mg kg^−1^ (available Se versus the abundance of ARGs, *P* = 2E−05) (Fig. 2d). The number of detected ARGs increased with increasing available Se content from 0.003 to 0.015 mg kg^−1^ (R^2^ = 0.38, *P* = 0.04) and decreased with available Se content from 0.015 to 0.14 mg kg^−1^ (R^2^ = 0.43, *P* = 0.01). The abundance of ARGs first increased with elevated available Se content ranging from 0.003 to 0.034 mg kg^−1^ (R^2^ = 0.67, *P* < 0.001) and then decreased with available Se content ranging from 0.034 to 0.14 mg kg^−1^ (R^2^ = 0.47, *P* = 0.03).

**Fig. 5:**
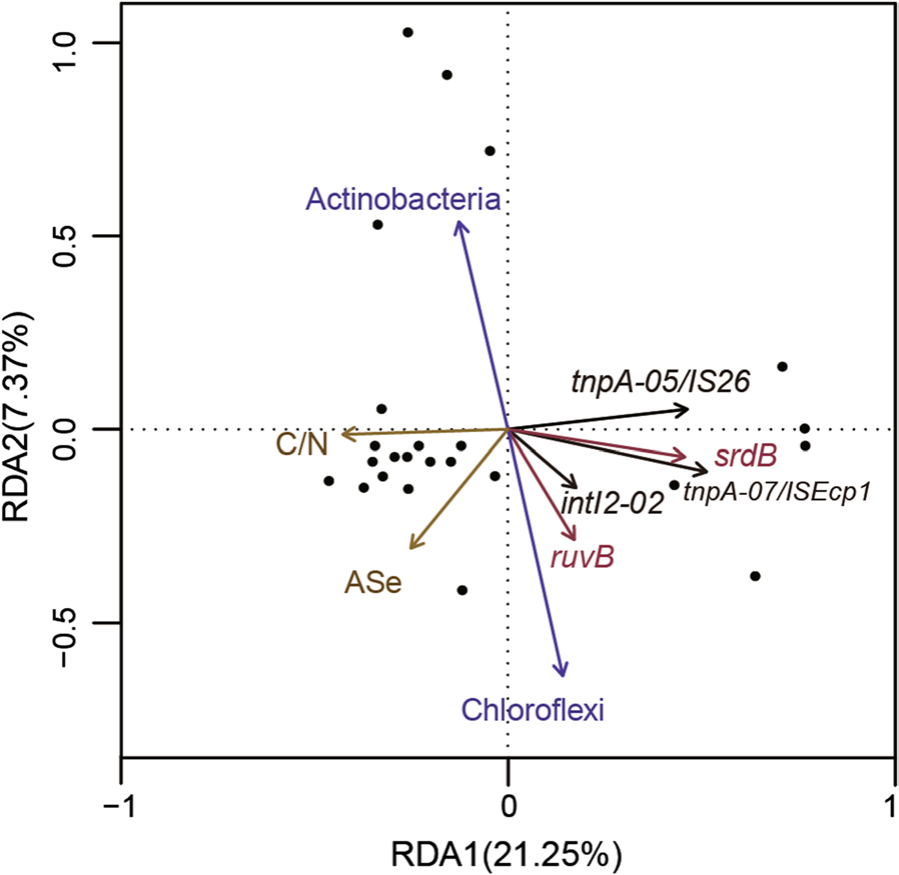
Redundancy analysis (RDA) of the significant soil chemical properties, bacterial phylum, MGEs, and Se resistance genes on the pattern of ARGs. C/N refers to the ratio of total soil carbon and total soil nitrogen. ASe refers to available Se content

Redundancy analysis (RDA) was used to examine the impact of environmental factors, Se resistance genes, MGEs, and bacterial community on the patterns of ARGs (Fig. 5). The RDA model explained 54% of the total variance of ARGs. Environmental factors, including the available Se content and C/N ratio, significantly affected the patterns of ARGs in the first and second canonical axes. The relative abundance of Se resistance genes, including *ruvB* and *srdB*, also significantly affected the patterns of ARGs. Actinobacteria and Chloroflexi were the two bacterial phyla that had significant effects on the pattern of ARGs. A few MGEs including *intI2-02*, *tnpA-05/IS26* and *tnpA-07/ISEcp1* had significant (*P* < 0.05) effects on the composition of ARGs.

**Fig. 6:**
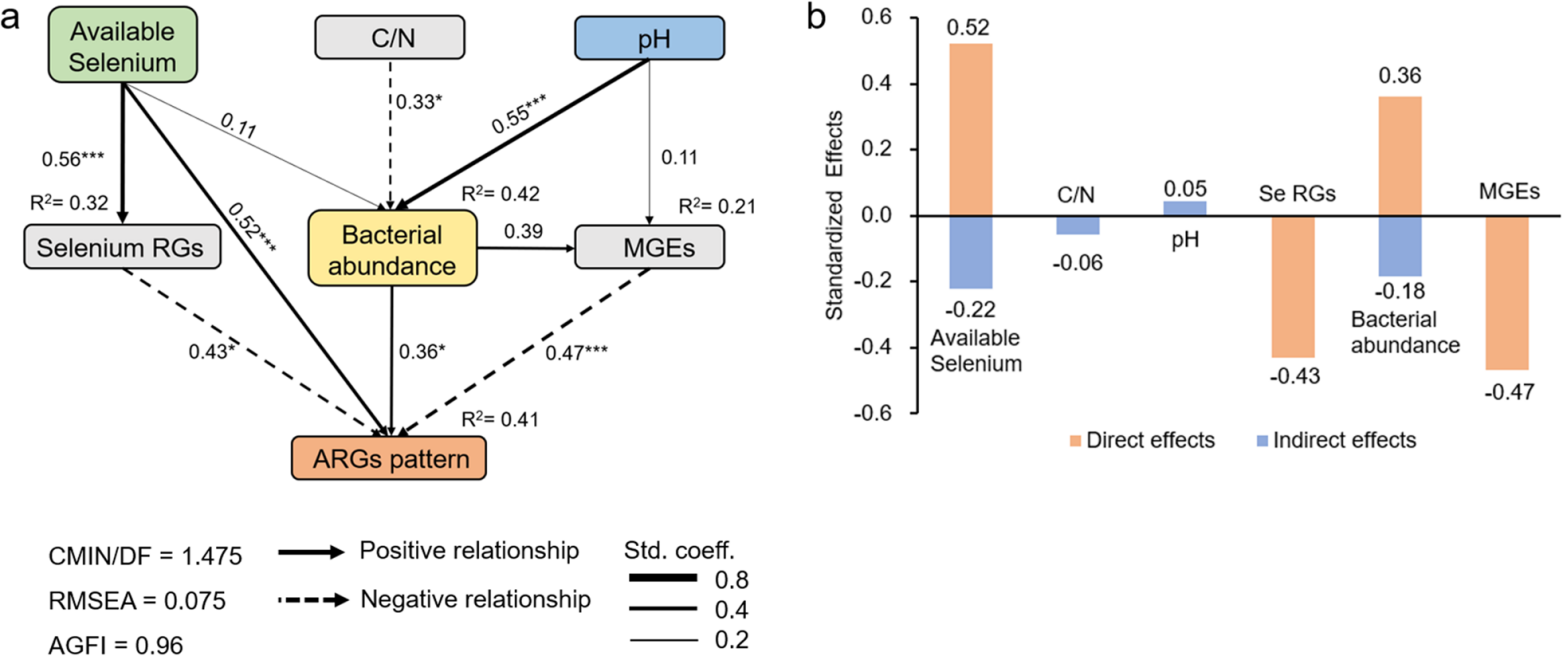
Structural equation models (SEM) showing relationships between chemical and biological parameters. **a** Direct and indirect effect of soil properties, selenium resistance genes, bacterial abundance, and MGEs on the pattern of ARGs. **b** The standardized total effects on ARGs R^2^ values are the explained proportion of variance. Significance levels of each path are indicated: *P < 0.05, **P < 0.01, ***P < 0.001. The hypothetical models fit the data well and were examined by CMIN/DF = 1.475, RMSEA = 0.075, AGFI = 0.96

Structural equation modeling (SEM) was established to integrate direct and indirect influences of variables on ARGs patterns (Fig. 6a, b). A Chi-square *P* value of 0.702 was obtained for the SEM, and it indicated that the model described the actual relationship among the variables (*P* > 0.05). The goodness of fit test showed that root mean square error of approximation (RMSEA) was 0.075, and adjusted goodness-of-fit index (AGFI) was 0.96. As shown in Fig. 6a, the overall variables explained 41% of the total variance of ARGs pattern. The model suggested that of the three edaphic variables (available Se, pH and C/N), available Se, with the highest standardized total effect of 0.299 (compared to 0.045) and -0.058 for pH and C/N, respectively), was the primary driving factor of the ARGs patterns (Fig. 6b). Furthermore, available Se content had a significant direct effect on ARGs patterns and also affected ARGs pattern through the indirect effects of Se resistance genes and bacterial abundance.

### Isolation and cultivation of Se resistant and antibiotic-resistant bacteria

**Fig. 7:**
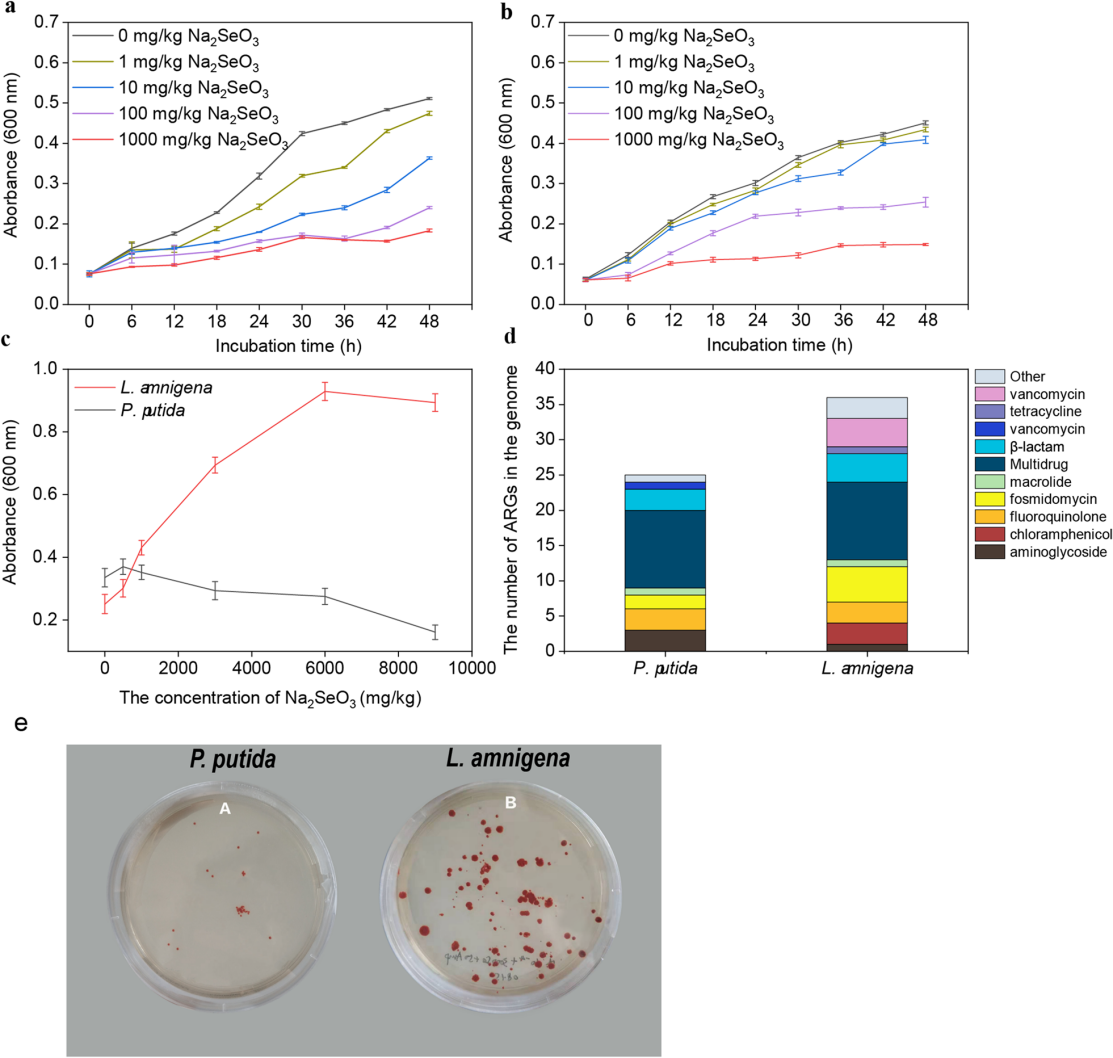
Effects of sodium selenite on the growth of total cultured bacteria originating from high Se content site (**a**) and low Se content site (**b**) over 48 h. Effects of different concentrations of sodium selenite on the growth of pure cultures after cultured for 48 h (**c**). Each value is the mean ± SD of three replicates. The number of putative ARGs in the genome of *P*. *putida* (low selenite resistance) and *L*. *amnigena* YTB01 (high selenite resistance) (**d**). The colonies of *P*. *putida* and *L*. *amnigena* YTB01 cultured for 48 h on TSB supplemented with 3000 mg kg^−1^ sodium selenite (**e**)

To further verify the correlation between Se content and ARGs, we used culture-based sodium selenite treatment to select Se resistant and antibiotic-resistant bacteria. The growth of total culturable bacteria was inhibited by sodium selenite (Fig. 7a, b). By comparison, cultures originating from the highest Se content site had a stronger ability to tolerate sodium selenite than those from the lowest Se content site. In addition, Se resistant and antibiotic-resistant bacteria were isolated on TSA plates amended with 50 μg mL^−1^ ampicillin and 3000 mg kg^−1^ sodium selenite from the highest and the lowest selenium content sites.

Subsequently, three colonies were selected from each of the two plates and were subsequently identified (using phylogenetic analysis of 16S rRNA gene sequences) as two bacteria, namely *Lelliottia amnigena* YTB01 (from highest selenium content site) and *Pseudomonas putida* (from lowest selenium content site). The sodium selenite susceptibility assay in TSB showed that sodium selenite had a dual effect on the growth of *Lelliottia amnigena* YTB01 and *Pseudomonas putida*. Specifically, the growth of *L. amnigena* YTB01 was promoted by sodium selenite concentration of up to 9000 mg kg^−1^. In contrast, the concentration of sodium selenite below 500 mg kg^−1^ promoted the growth of *Pseudomonas putida*, while sodium selenite concentrations higher than 500 mg kg^−1^ inhibited the growth of *Pseudomonas putida* (Fig. 7c). The two isolates grew on TSA supplemented with 3000 mg kg^−1^ sodium selenite (Fig. 7e). The colonies of *Lelliottia amnigena* YTB01 on TSA were bigger in size and much more in numbers than those of *Pseudomonas putida*. These results demonstrated that the growth of total culturable bacteria was inhibited by sodium selenite and suggested that the two strains differed in their abilities to tolerate sodium selenite.

### The genome analyses of Se-resistant bacteria

**Fig. 8:**
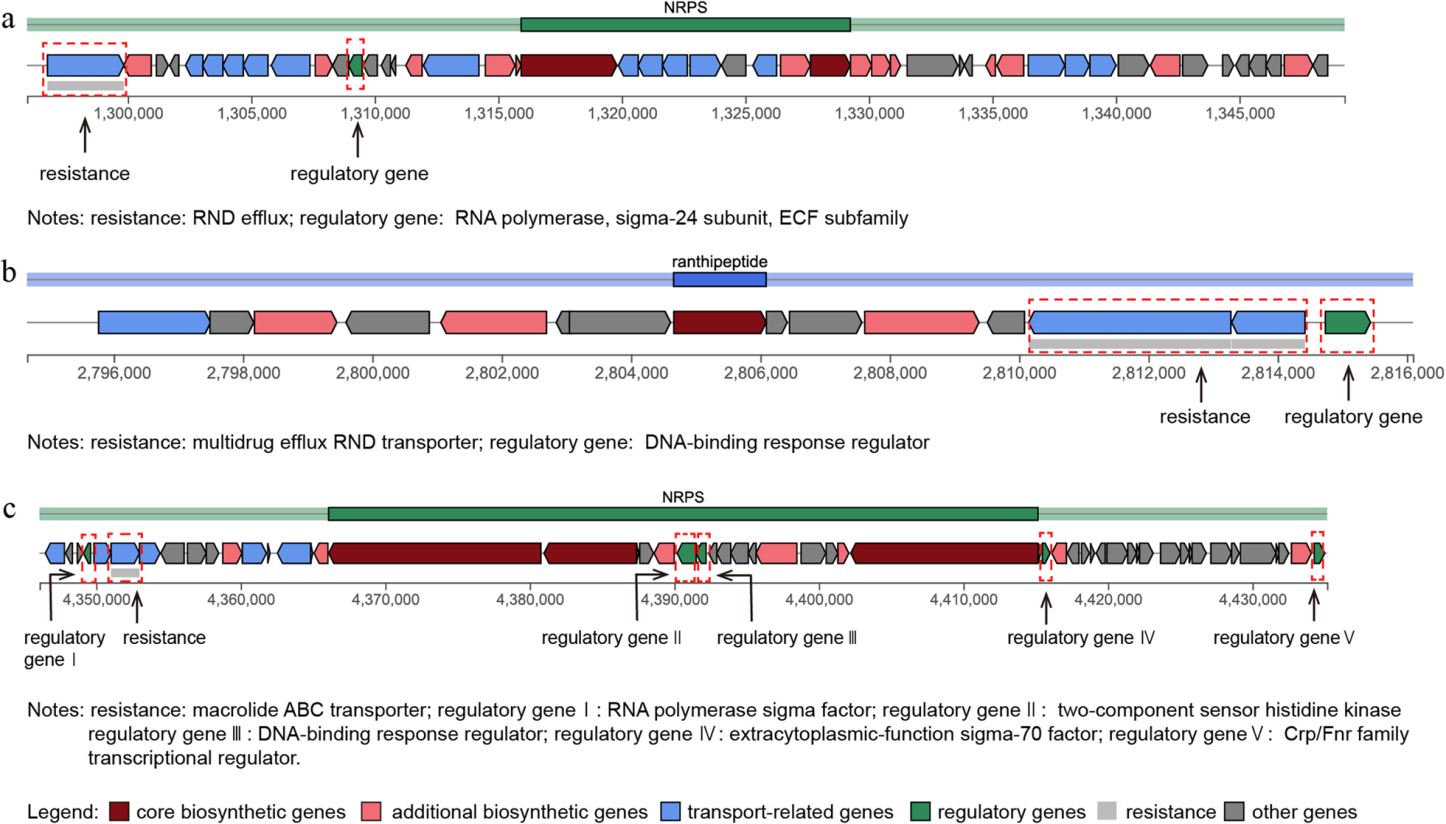
Schematic representation of the important structures related to the regulation of antibiotics resistance genes expression in two typical secondary metabolism BGCs extracted from the genomes of the two isolates. **a** In the genome of *Lelliottia amnigena* isolation from the highest Se content site. **b** and **c** In the genome of *Pseudomonas putida* isolation from the lowest Se content site

Next, we sequenced the whole genome of the Se resistant *Lelliottia amnigena* YTB01 and *Pseudomonas putida* in order to investigate the co-occurrence pattern of Se resistance genes and ARGs. The genome of *Lelliottia amnigena* YTB01 is 4,941,692 bp in size and has a G+C content of 54.43%, including 4605 coding genes and 141 non-coding RNA (ncRNA) (Additional file 4: Fig. S4a). The genome annotation revealed various putative proteins involved in Se resistance and antibiotic resistance (Additional file 5: Material 1), including multidrug resistance protein *EmrD*, fosfomycin resistance protein *FosA*, tellurite resistance proteins *TehA/TehB*, and selenium resistant proteins *RuvA/RuvB*. The genome of *Pseudomonas putida* is 6,287,193 bp in size and has a G+C content of 62.34%, including 5,624 coding genes and 139 ncRNA (Additional file 4: Fig. S4b and Additional file 6: Material 2). Importantly, the number of ARGs and Se resistance genes in the genome of *Lelliottia amnigena* YTB01 were 38 and 11, respectively, which were more than the 25 and 9, respectively, in the genome of *P. putida*. This observation is striking given that the complete genome of *Lelliottia amnigena* YTB01 isolated from the highest Se content site is smaller than the genome of *Pseudomonas putida* isolated from the lowest Se content site (Fig. 7d). Furthermore, in the genome of *Lelliottia amnigena* YTB01, a hypothetical antibiotic resistance RND efflux gene was identified, and an RNA polymerase was predicted as a regulatory gene (Fig. 8a). In the genome of *Pseudomonas putida*, two gene clusters both harboring resistance and regulator genes were predicted. In one cluster, a multidrug efflux RND transporter was predicted as a resistance gene downstream, followed by a DNA-binding response regulator (Fig. 8b). In another cluster, a macrolide ABC transporter was predicted as a resistance gene while five regulatory genes were detected (Fig. 8c).

## Discussion

### Soil Se induces the dual effects of ARGs pattern

Soil is a vast reservoir of ARGs, and the migration of ARGs in the natural environment might pose a public health threat [64, 65]. In our study, we detected a high diversity and abundance of ARGs in seleniferous forest soils and found a significant dual effect between available Se and the diversity or the abundance of ARGs with available Se thresholds of 0.015 mg kg^−1^ and 0.034 mg kg^−1^. These results indicated that the ARGs pattern are different below and above the thresholds. Interestingly, the two thresholds are not consistent, suggesting the diversity and abundance of ARGs respond differently to Se stress. Considering that the abundance of ARGs is more relevant for transferring ARGs between non-pathogenic and pathogenic bacteria than the diversity of ARGs, the threshold of 0.034 mg kg^−1^ available Se is further discussed in this study. The role of metal(loid)s as co-selective pressures driving ARGs distribution has been widely demonstrated [66–68]. The present study showed that Se can induce a dual effect on ARGs pattern, unlike the positive correlation between metal(loid)s and ARGs reported in several previous studies [10, 69]. A possible explanation is the varied co-occurrence mechanisms between different metal(loid)s and ARGs. In addition, the concentration of the only two detected antibiotics is quite low and is not correlated with the abundance of ARGs, indicating the weak influence of anthropogenic activities in this area. Therefore, we speculate that antibiotics residues are not the drivers of ARGs changes in this area. Altogether, the data regarding an association between available Se and ARGs demonstrate that the diversity and abundance of ARGs might be increased in moderate Se content soils but decreased in high Se content soils. Given that the available Se content of most soils in the world is below the threshold of 0.034 mg kg^−1^, the elevated soil Se content caused by Se-enrich agricultural farming can potentially lead to the enrichment of ARGs.

### Bacterial community is an important contributor to the variance of ARGs

The bacterial community is the carrier of ARGs and is widely considered to be an important contributor to the variance of ARGs [70]. In this study, we found that available Se had a dual effect on the abundance of the bacterial community, and this effect was consistent with the correlation between available Se content and the abundance of ARGs. This observation indicates that bacterial communities may play a distinct and important role in influencing the ARGs pattern, which was further corroborated by SEM. The explanation for the observed dual effect may be that moderate content of Se is essential and beneficial for bacterial proliferation [71], which can lead to an increase in bacterial abundance, thereby enriching their associated ARGs. Previous evidence from culturable bacteria also suggested that Se-enriched bacteria are more resistant to antibiotics than those not treated with Se [21]. However, excessive Se, especially soluble and toxic selenite, can inhibit the growth of bacteria [13, 71] and thus reduce the corresponding ARGs’ abundance.

Further evidence showed that the dual effect of available Se on bacterial abundance was comprehensively influenced by *ruvB*, *recG* and *tehB*. Hence, it was more likely that the toxicity of Se to bacterial cells will enhance with the increase of available Se concentration, and the abundance of DNA damage repair genes such as *ruvB* and *recG* will raise [19]. When the available Se concentration is lower than the threshold (0.08 mg kg^−1^), the abundance of *tehB* gene will increase and involves Se methylation. The methylation is also able to detoxify Se [20], which contributes to cell proliferation, thus increasing bacterial abundance. On the contrary, when the available Se is higher than the threshold, the abundance of *tehB* decreases, which weakens Se-detoxication via Se methylation. Interestingly, complete genome analyses verified the presence of both DNA repair (*ruvB* and *recG*) and Se methylation (*tehB*) genes in the genome of both *Lelliottia amnigena* YTB01 and *Pseudomonas putida.*

In this study, HGT may not be the main mechanism for the transfer of ARGs in the forest soils. First, *Intl1*, a gene responsible for HGT or acquisition of ARGs in environmental microorganisms [28], had a low frequency and abundance in this study. Interestingly, this is consistent with previous reports that the abundance of *intl1* is relatively low in environments with minimal anthropogenic impact [5, 72]. Second, a weak correlation between the total abundance of MGEs and ARGs suggests that HGT is not the main mechanism contributing to ARG transfer. Rather, the fact that Se may indirectly mediate the migration of ARGs by regulating the abundance of bacterial communities, indicated that vertical genetic transfer is the dominant mechanism of ARG migration in forest soils.

### Co-selection of Se resistance genes and ARGs

An increasing number of studies have demonstrated that metal(loid)s contamination may promote the spread of ARGs through co-selection effect [8–10]. Importantly, *ruvB* (involved in the repairing of DNA damage) and *srdB* (selenate reductase subunit B) had a significant effect on the pattern of ARGs, suggesting that Se pressure may influence the co-selection of Se resistance genes and ARGs. The co-selection effect is due to the coupling of resistance mechanisms against metal(loid)s and antibiotics [73, 74]. Co-occurrence network analysis revealed a close correlated cluster among ARGs, Se resistance genes, and MGEs, suggesting that ARGs and Se resistance genes may co-occur on the same genetic element or is co-present within the same cell.

Another potential mechanism for Se detoxication is the reduction of Se oxyanions to elemental Se. For example, bacteria can reduce soluble and higher toxic Se (IV) and Se (VI) oxyanions to less toxic Se (0) and bind the elemental Se to the selenate reductase A protein to form a Se nanosphere, which is subsequently exported to the extracellular environment [75, 76]. This was also observed in the culturable bacteria experiment where the two isolates transformed colorless sodium selenite to red elemental Se. The active efflux of the elemental Se may be accompanied by the efflux of antibiotics from the cell to the extracellular environment. This potential co-efflux of Se and antibiotics from the cell is considered a cross-resistance mechanism [77]. However, further studies are required to investigate the hypothesis.

Using culture-based methods, we confirmed that the growth of total cultured bacteria was inhibited by sodium selenite. This observation was in contrast to the observed correlation between available Se and the abundance of the bacterial community (Fig. 3a). The reason might be that the cultured bacteria only accounted for less than 1% of the total bacterial community, and 16S rRNA gene analysis cannot differentiate between dead and alive bacteria. Moreover, the total bacterial cultures from the higher soil Se content site had a stronger ability to tolerate sodium selenite than those from the lower soil Se content site, and this observation was consistent with the results of pure cultures. In addition, the numbers of putative ARGs on the genome of *Lelliottia amnigena* YTB01 (high Se tolerance) were much more than those of *Pseudomonas putida* (low Se tolerance), which indicated that Se may have cooperative associations with bacterial ARGs. Altogether, these observations supported that Se influences the pattern of ARGs in Se-rich forest soil. However, compared with the environmental concentration, the concentration of sodium selenite used to select culturable bacteria may be too high; this constitutes a limitation of this study, and further studies are required to explore lower concentration of sodium selenite in this regard.

The genomic mining of both *Lelliottia amnigena* YTB01 and *Pseudomonas putida* indicated that some multidrug efflux RND transporter and macrolide ABC transporter harbored dual functions as transporter protein and resistance genes, regulated by RNA polymerase and DNA-binding response regulator, respectively. Altogether, these gene repertoire confirms the insights obtained from the metagenomic on the mechanism of Se detoxification in the sampled soils. Furthermore, it suggests possible co-selective or co-regulatory resistance mechanisms for transporting both Se and antibiotics to the extracellular space. In summary, high Se pressure may dominantly drive the co-selection of Se resistance and antibiotic resistance through cross-resistance and co-regulatory resistance), thereby influencing ARGs diversity and abundance.

## Conclusion

In this study, by investigating soils with a wide range of Se contents, we provided evidence that the diversity and abundance of ARGs are enhanced under moderate Se content but inhibited under severe Se pressure. In addition, the methylation of Se (mediated by *tehB*) and the repair of DNA damages (mediated by *ruvB* and *recG*) are the dominant mechanisms of Se resistance in the study area, and such Se resistance may influence ARGs pattern. Bacterial communities also play an important role in mediating the dual effects of soil Se on the distribution of ARGs. Furthermore, the cultured high Se-tolerant bacteria possessed more ARGs on the genome than the low Se-tolerant bacteria. Remarkably, the world average soil Se content is lower than the threshold of 0.034 mg kg^−1^; therefore, elevated soil Se content may facilitate the spread of antibiotic resistance.

## Supplementary Information

**Additional file 1: Fig. S1**. Heatmap of the relative abundance of ARGs. The column is labeled with the content of available Se content of each sampling from low to high. The row is the ARGs detected in each sample and grouped according to antibiotic resistance type.

**Additional file 2: Fig. S2**. Relative abundance of the bacterial community at the phylum level.

**Additional file 3: Fig. S3**. Co-occurrence network analysis showing the correlation between ARGs, MGEs, and Se resistance genes (R > 0.8, *P* < 0.05). Node size indicates connectivity, and the color of nodes is grouped by modularity class.

**Additional file 4: Fig. S4**. The genome circles graphs of Lelliottia amnigena YTB01 (a) and Pseudomonas putida (b). From outer to inner: 1: Genome Size; 2: Forward Strand Gene, colored according to the cluster of orthologous groups (COG) classification; 3: Reverse Strand Gene, colored according to the cluster of orthologous groups (COG) classification; 4: Forward Strand ncRNA; 5: Reverse Strand ncRNA; 6: repeat; 7: GC; 8: GC-SKEW.

**Additional file 5: Material 1**. The genome annotation of various putative proteins involved in Se resistance and antibiotic resistance of *Lelliottia amnigena* YTB01.

**Additional file 6: Material 2**. The genome annotation of various putative proteins involved in Se resistance and antibiotic resistance of *Pseudomonas putida*.

**Additional file 7: Table S1**. Sampling site locations and soil chemical properties. **Table S2**. Primer sequences and quantitative PCR reaction conditions of 16 selenium resistance genes. **Table S3**. Pearson correlation between soil chemical properties. “*” significant correlations at *P* < 0.05; “**” Significant correlations at *P* < 0.001. **Table S4**. The detected concentration of antibiotic residues (µg kg^−1^). **Table S5**. The absolute copy numbers of selenium resistance genes (copies g^−1^).

**Additional file 8: Text S1**. Determination of total and available Se contents of soil.

## Data Availability

The datasets generated during the current study are available in the NODE repository (https://www.biosino.org/node/index), including all 16S rRNA gene sequences (Accession No. OEP001738) and the complete genome sequences of two isolates (Accession No. OEP001741).

## Abbreviations

ARGs: Antibiotic resistance genes
BGCs: Biosynthetic gene clusters
HGT: Horizontal gene transfer
MGEs: Mobile genetic elements
RDA: Redundancy analysis
SEM: Structural equation modeling
VGT: Vertical gene transfer
ncRNA: Non-coding RNA
MLSB: Macrolide lincosamide streptogramin_b
OTUs: Operational taxonomic units
SOC: Soil organic carbon
TN: Total nitrogen
TCN: Tetracycline
DOX: Doxycycline hydrochloride
OLF: Ofloxacin
ENR: Enrofloxacin
AMX: Amoxicillin
CA: Clavulanic acid
KAN: Kanamycin
ENY: Erythromycin
