# Supplementary material for: Natural selenium stress influences the changes of antibiotic resistome in seleniferous forest soils
Source: Environ Microbiome. 2022 May 15;17:26. doi: 10.1186/s40793-022-00419-z (PMC9107767; doi:10.1186/s40793-022-00419-z)
Supplement: Supplementary file 1 — Additional file 1: Fig. S1. Heatmap of the relative abundance of ARGs. The column is labeled with the content of available Se content of each sampling from low to high. The row is the ARGs detected in each sample and grouped according to antibiotic resistance type. [file 40793_2022_419_MOESM1_ESM.pdf]

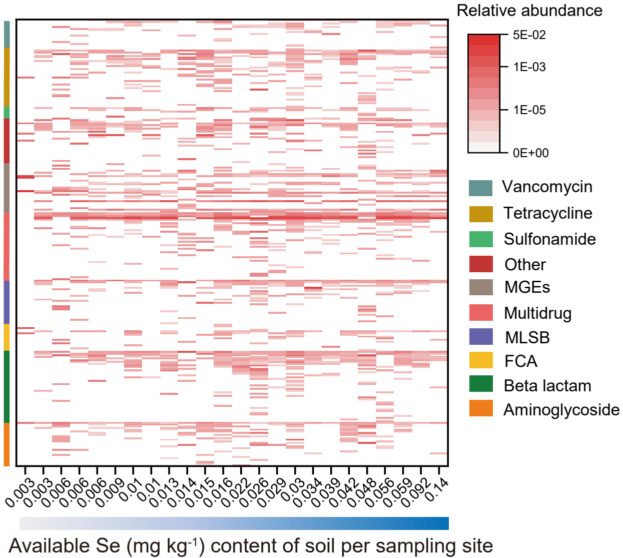

Relative abundance
5E-02
1E-03
1E-05
0E+00
Vancomycin
Tetracycline
Sulfonamide
Other
MGEs
Multidrug
MLSB
FCA
Beta lactam
Aminoglycoside
0.003
0.003
0.006
0.006
0.006
0.009
0.01
0.01
0.013
0.014
0.015
0.016
0.022
0.026
0.029
0.03
0.034
0.039
0.042
0.048
0.056
0.059
0.092
0.14
Available Se (mg kg-1) content of soil per sampling site
