# Supplementary figures and images for: Natural selenium stress influences the changes of antibiotic resistome in seleniferous forest soils
Source: Environ Microbiome. 2022 May 15;17:26. doi: 10.1186/s40793-022-00419-z (PMC9107767; doi:10.1186/s40793-022-00419-z)

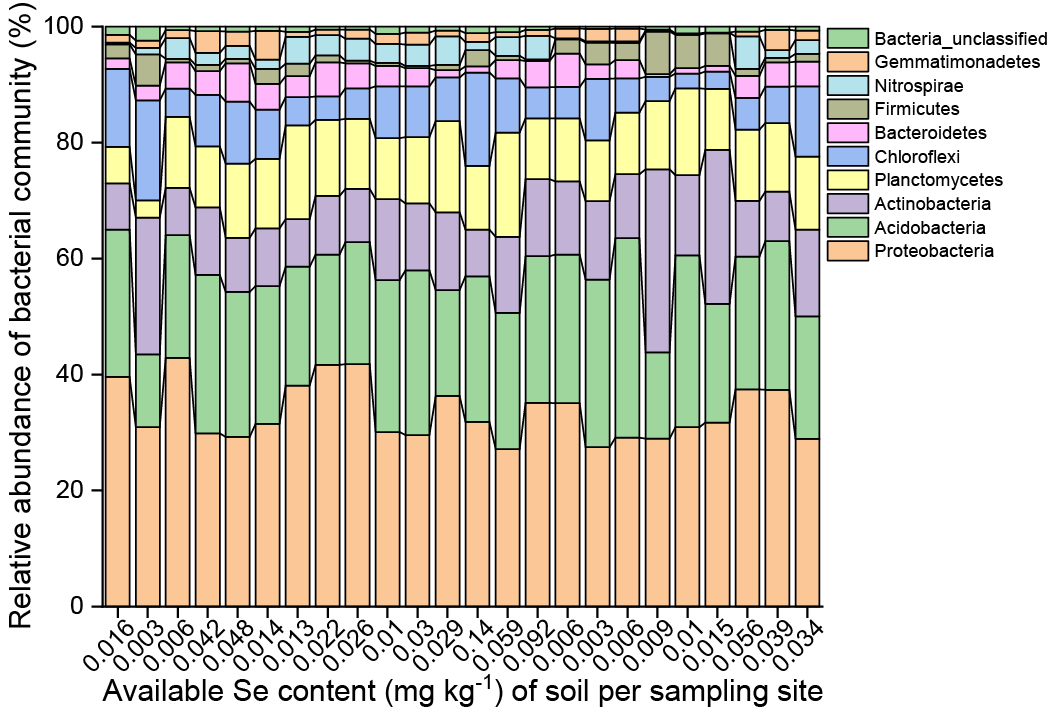

Supplement: Supplementary file 2 — Additional file 2: Fig. S2. Relative abundance of the bacterial community at the phylum level. [file 40793_2022_419_MOESM2_ESM.tif]

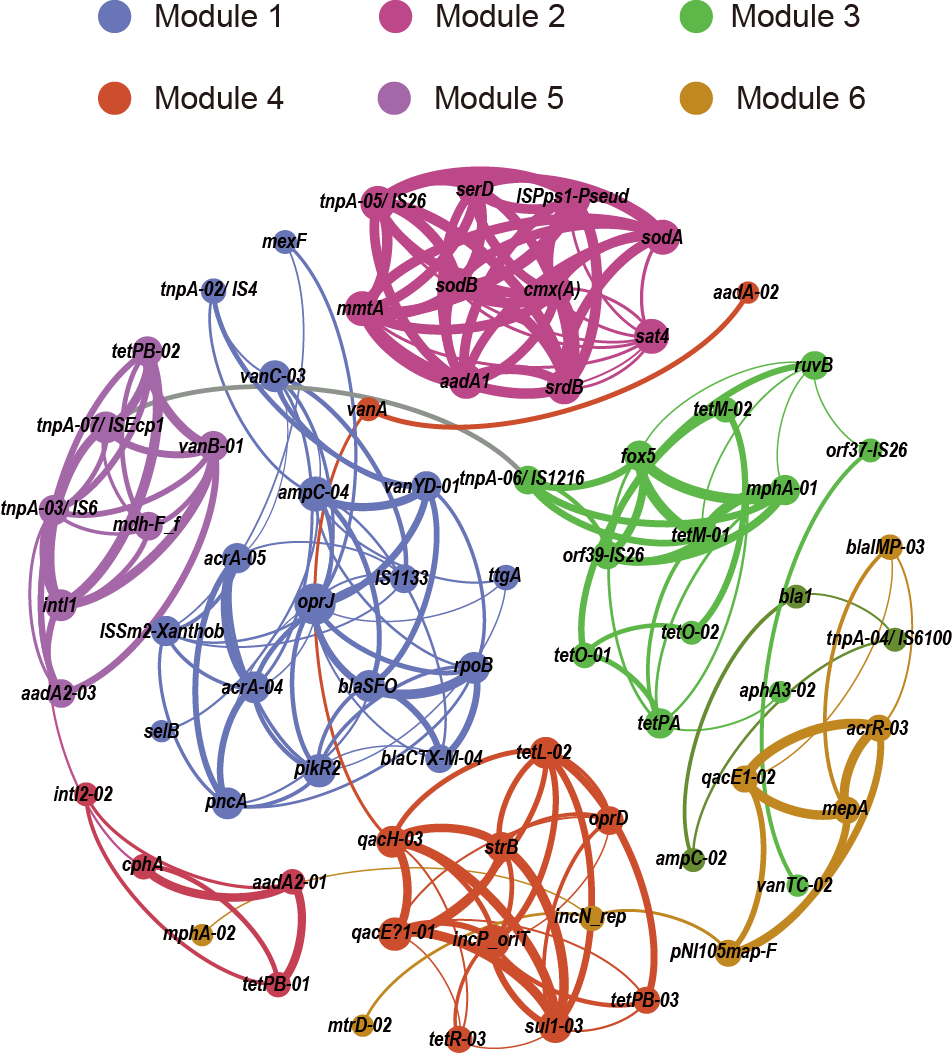

Supplement: Supplementary file 3 — Additional file 3: Fig. S3. Co-occurrence network analysis showing the correlation between ARGs, MGEs, and Se resistance genes (R > 0.8, P < 0.05). Node size indicates connectivity, and the color of nodes is grouped by modularity class. [file 40793_2022_419_MOESM3_ESM.tif]

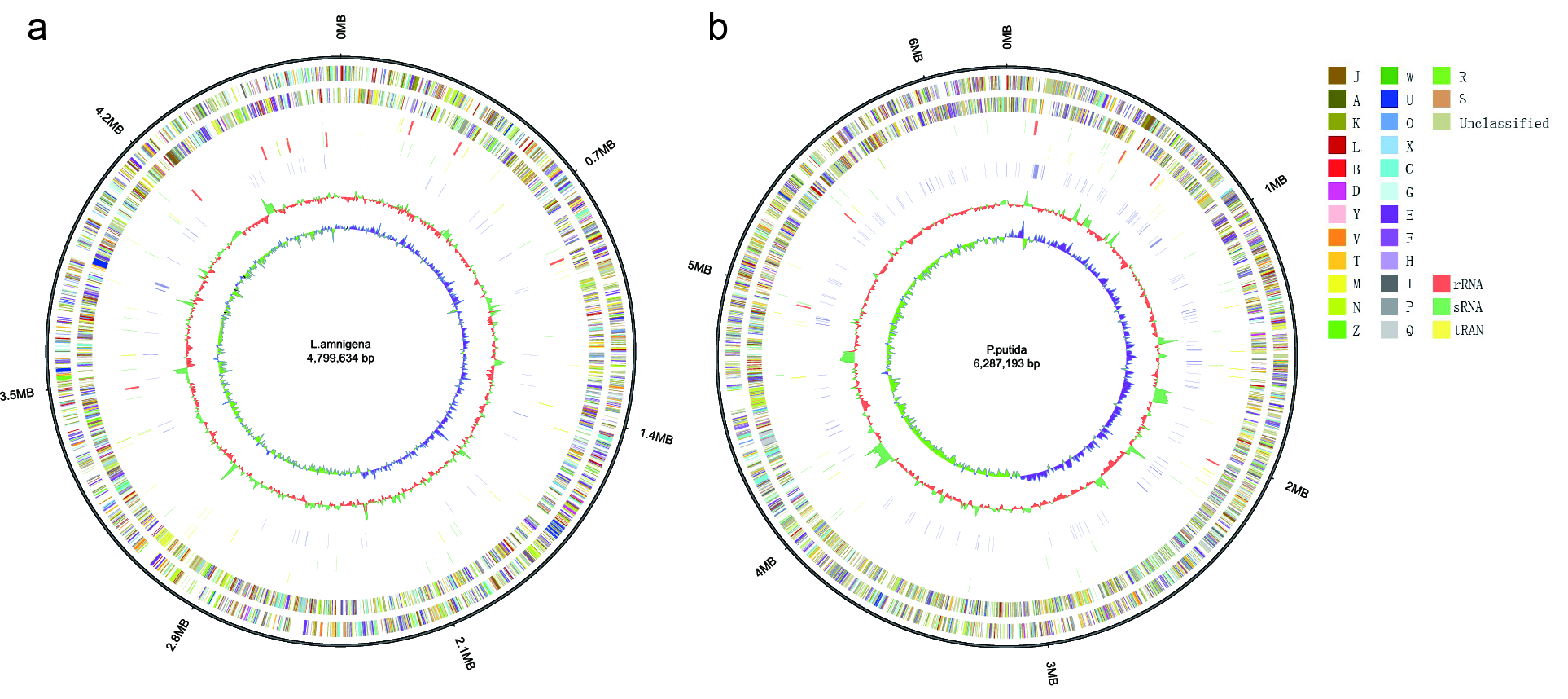

Supplement: Supplementary file 4 — Additional file 4: Fig. S4. The genome circles graphs of Lelliottia amnigena YTB01 (a) and Pseudomonas putida (b). From outer to inner: 1: Genome Size; 2: Forward Strand Gene, colored according to the cluster of orthologous groups (COG) classification; 3: Reverse Strand Gene, colored according to the cluster of orthologous groups (COG) classification; 4: Forward Strand ncRNA; 5: Reverse Strand ncRNA; 6: repeat; 7: GC; 8: GC-SKEW. [file 40793_2022_419_MOESM4_ESM.tif]
