# Supplementary material for: Natural selenium stress influences the changes of antibiotic resistome in seleniferous forest soils
Source: Environ Microbiome. 2022 May 15;17:26. doi: 10.1186/s40793-022-00419-z (PMC9107767; doi:10.1186/s40793-022-00419-z)
Supplement: Supplementary file 7 — Additional file 7: Table S1. Sampling site locations and soil chemical properties. Table S2. Primer sequences and quantitative PCR reaction conditions of 16 selenium resistance genes. Table S3. Pearson correlation between soil chemical properties. “*” significant correlations at P < 0.05; “**” Significant correlations at P < 0.001. Table S4. The detected concentration of antibiotic residues (µg kg−1). Table S5. The absolute copy numbers of selenium resistance genes (copies g−1). [file 40793_2022_419_MOESM7_ESM.docx]

Table S1 Locations and soil chemical properties of 24 sites.

| Site | Sample | Latitude/Longitude | pH | TSC | TSN | C/N | TP | TK | Total Se | Available Se |
| --- | --- | --- | --- | --- | --- | --- | --- | --- | --- | --- |
|  |  |  |  | (g C kg^-1^) | (g N kg^-1^) |  | (g P kg^-1^) | (mg K kg^-1^) | (mg kg^-1^) | (mg kg^-1^) |
| 1 | CJB | 30.29°E/108.96°N | 6.01±0.02 | 10.31±0.12 | 1.02±0.02 | 10.11±0.01 | 0.55±0.02 | 16.21±0.13 | 0.08±0.01 | 0.003±0.0001 |
| 2 | HCT | 30.47°E/109.64°N | 5.68±0.01 | 12.81±0.20 | 1.09±0.07 | 11.75±0.02 | 0.39±0.01 | 14.46±0.11 | 2.24±0.01 | 0.003±0.0001 |
| 3 | XQC | 30.29°E/108.96°N | 6.56±0.01 | 14.66±0.14 | 1.39±0.02 | 10.55±0.02 | 0.38±0.01 | 15.03±0.15 | 0.69±0.02 | 0.006±0.0002 |
| 4 | XJW | 30.23°E/109.02°N | 6.09±0.02 | 12.88±0.25 | 1.08±0.01 | 11.93±0.03 | 0.6±0.03 | 14.61±0.20 | 0.06±0.01 | 0.006±0.0001 |
| 5 | ZJW | 29.98°E/109.47°N | 6.05±0.03 | 12.62±0.20 | 1.21±0.02 | 10.43±0.02 | 0.64±0.01 | 15.97±0.01 | 0.08±0.02 | 0.006±0.0001 |
| 6 | DLT | 30.27°E/109.44°N | 6.1±0.01 | 31.24±0.28 | 2.73±0.01 | 11.44±0.02 | 0.93±0.02 | 10.51±0.03 | 0.60±0.01 | 0.009±0.0002 |
| 7 | SKQ | 30.27°E/109.50°N | 6.64±0.02 | 19.55±0.16 | 1.86±0.04 | 10.51±0.01 | 0.65±0.01 | 11.77±0.05 | 0.66±0.03 | 0.01±0.0012 |
| 8 | WJW | 30.27°E/109.50°N | 6.86±0.01 | 31.81±0.10 | 2.15±0.01 | 14.8±0.02 | 0.7±0.03 | 11.36±0.11 | 0.32±0.01 | 0.01±0.0004 |
| 9 | DDW | 30.26°E/109.05°N | 5.99±0.02 | 39.35±0.35 | 3.06±0.02 | 12.86±0.02 | 0.64±0.03 | 14.91±0.14 | 2.90±0.01 | 0.013±0.0002 |
| 10 | SBY | 30.15°E/109.64°N | 6.45±0.01 | 12.03±0.05 | 1.06±0.01 | 11.35±0.01 | 0.62±0.01 | 15.73±0.13 | 0.53±0.02 | 0.014±0.0011 |
| 11 | SDH | 30.65°E/110.35°N | 6.1±0.02 | 31.83±0.27 | 2.81±0.01 | 11.33±0.02 | 0.8±0.02 | 17.24±0.03 | 0.57±0.01 | 0.015±0.0002 |
| 12 | YTB2 | 30.37°E/110.04°N | 6.72±0.02 | 15.34±0.32 | 1.37±0.03 | 11.2±0.03 | 0.59±0.02 | 17.03±0.18 | 1.66±0.03 | 0.016±0.0004 |
| 13 | SH | 30.17°E/109.60°N | 6.2±0.01 | 36.68±0.22 | 3.22±0.02 | 11.39±0.02 | 0.63±0.01 | 14.93±0.10 | 2.36±0.01 | 0.022±0.0002 |
| 14 | LHB | 29.98°E/109.47°N | 6.01±0.03 | 32.25±0.28 | 2.82±0.01 | 11.44±0.01 | 0.57±0.01 | 16.92±0.15 | 1.20±0.02 | 0.026±0.0001 |
| 15 | SQZ | 30.51°E/109.62°N | 6.49±0.01 | 26.5±0.26 | 2.36±0.02 | 11.23±0.03 | 0.6±0.02 | 16.24±0.13 | 0.56±0.01 | 0.029±0.0002 |
| 16 | JYZ | 30.29°E/110.03°N | 6.68±0.01 | 21.38±0.13 | 2.03±0.01 | 10.53±0.01 | 0.68±0.05 | 12.85±0.12 | 0.57±0.01 | 0.030±0.0001 |
| 17 | MJT | 30.05°E/110.03°N | 6.09±0.01 | 31.77±0.10 | 2.93±0.03 | 10.84±0.02 | 0.68±0.04 | 13.7±0.20 | 4.44±0.01 | 0.034±0.0002 |
| 18 | YTY | 30.46°E/110.33°N | 6.37±0.01 | 24.66±0.23 | 2.45±0.02 | 10.07±0.01 | 0.64±0.01 | 18.99±0.11 | 4.77±0.01 | 0.039±0.0001 |
| 19 | SZP | 30.28°E/108.96°N | 6.75±0.01 | 13.31±0.22 | 1.28±0.01 | 10.4±0.01 | 0.6±0.02 | 17.67±0.24 | 0.73±0.02 | 0.042±0.0001 |
| 20 | LZH | 30.44°E/110.28°N | 6.89±0.02 | 15.84±0.21 | 1.53±0.02 | 10.35±0.02 | 0.59±0.02 | 16.47±0.05 | 0.55±0.01 | 0.048±0.0002 |
| 21 | YTB1 | 30.37°E/110.04°N | 6.38±0.02 | 29.41±0.25 | 2.56±0.01 | 11.49±0.01 | 0.65±0.01 | 14.01±0.02 | 9.85±0.01 | 0.056±0.0001 |
| 22 | MJW | 30.45°E/110.32°N | 7.52±0.02 | 37.39±0.37 | 2.97±0.02 | 12.59±0.01 | 0.67±0.02 | 10.02±0.04 | 3.43±0.01 | 0.059±0.0002 |
| 23 | TZY1 | 30.48°E/109.83°N | 7.13±0.01 | 38.55±0.35 | 3.59±0.01 | 10.74±0.02 | 0.61±0.03 | 11.21±0.31 | 13.48±0.01 | 0.092±0.0001 |
| 24 | TZY2 | 30.61°E/110.01°N | 6.9±0.01 | 30.19±0.48 | 3.02±0.01 | 10±0.01 | 0.55±0.01 | 12.76±0.34 | 20.65±0.01 | 0.140±0.0001 |

Notes: TC, soil total carbon; TN, soil total nitrogen; C/N, carbon and nitrogen ratio; TP, soil total phosphorus; TK, soil total potassium; T Se, soil total selenium; A Se, soil available selenium.

Table S2 Primer sequences and quantitative PCR reaction conditions of 16 selenium resistance gene.

| Target gene | Gene Bank accession  number | Amplicon  size(bp) | Primer Sequences | TM  value | Annealing  temperature (℃) | Reference |
| --- | --- | --- | --- | --- | --- | --- |
| *mmtA* | AY569977.1 | 304 | F: 5’-CCGGTATCCGCCACGCACTG-3’ | 65.0 | 56 | [34] |
|  |  |  | R: 3’-GGAAACCCGCCTGCTCCAGAA-5, | 63.0 |  |  |
| *SelA* | NC_000913.3 | 301 | F: 5’-TTCGTATTCCCGATGTTATGC-3’ | 52.2 | 56 | [35] |
|  |  |  | R: 3’-GCCACCAGTTCCGCTTC-5’ | 57.5 |  |  |
| *SelB* | NC_000913.3 | 304 | F: 5’-AGAGCAGATTAACCGTGGCG-3’ | 57.9 | 58 | [35] |
|  |  |  | R: 3’-GCAATACCAGGCGGTCGTT-5’ | 58.7 |  |  |
| *SerA* | AJ007744.1 | 317 | F: 5’-CGCCAACGGCATCGAGGTCA-3’ | 64.0 | 58 | [36] |
|  |  |  | R: 3’-CCGATCAGGGTGAGGATGAGGA-5’ | 61.1 |  |  |
| *SerB* | AJ007744.1 | 324 | F: 5’-TCCCGAACAATCATTACTTCTACCTG-3’ | 56.0 | 58 | [36] |
|  |  |  | R: 3’-GCACTTCTCCGACGTGCCTTT-5’ | 60.9 |  |  |
| *SerC* | AJ007744.1 | 317 | F: 5’-ATCGTCGGCACCGCCCTTAC-3’ | 63.6 | 56 | [36] |
|  |  |  | R: 3’-GCGTGGTGTCCTTGCCATTCA-5’ | 61.3 |  |  |
| *SerD* | AJ007744.1 | 360 | F: 5’-GAGTACCTCGCCGCCTTCG-3’ | 61.9 | 58 | [36] |
|  |  |  | R: 3’-TTCCAGTTCGGTGACCAGATGAT-5’ | 58.1 |  |  |
| *srdA* | AB534554.1 | 313 | F: 5’-GGCTGAATGGGCAGAAT-3’ | 52.5 | 56 | [37] |
|  |  |  | R: 3’-ACCGCCAGTATTAGAAAT-5’ | 46.9 |  |  |
| *srdB* | AB534554.1 | 303 | F: 5’-GCCTCCCGGTGTTGTTT-3’ | 56.1 | 56 | [37] |
|  |  |  | R: 3’-CACCATACGGGCAAGCA-5’ | 55.8 |  |  |
| *srdC* | AB534554.1 | 297 | F: 5’-TGGCTCATTATTTGGTGTA-3’ | 47.5 | 56 | [37] |
|  |  |  | R: 3’-TAGGGTTCCTAATTCCTTT-5’ | 46.2 |  |  |
| *ubiE* | AY426747.1 | 309 | F: 5’-CTGGCTAAAGCAGGATATGAGGTAA-3’ | 56.0 | 56 | [34] |
|  |  |  | R:3’-CAGCGGTTGGTCCGAGAAT-5’ | 58.1 |  |  |
| *recG* | NC_002516.2 | 348 | F: 5’-CGGAACTGGCCCGCGATTAC-3’ | 62.3 | 56 | [16] |
|  |  |  | R:3’-GCCGAGGTTGGCAAGGAAGC-5’ | 63.0 |  |  |
| *ruvB* | NC_002516.2 | 368 | F: 5’-ATCGCCAATCGCCTGCTG-3’ | 59.4 | 58 | [16] |
|  |  |  | R: 3’-GTGCCGCTCCTCGCTCAA-5’ | 61.9 |  |  |
| *tehB* | NC_000913.3 | 292 | F: 5’-GCCATGAGTATCGCCAACG-3’ | 56.9. | 58 | [17] |
|  |  |  | R: 3’-ATCCATCGCCGCCACAA-5’ | 58.0 |  |  |
| *sodB* | EU900460.1 | 343 | F: 5’-ACACCTTCTACTGGAACTGCCTGAG-3’ | 60.7 | 56 | [15] |
|  |  |  | R: 3’-GAGCCGTCGGCCTTCTTCA-5’ | 60.9 |  |  |
| *sodA* | NC_000913.3 | 584 | F: 5’-CCTGCCGGTTGAAGAGC-3’ | 57.2 | 59 | [15] |
|  |  |  | R: 3’-CCTGCCGGTTGAAGAGC-5’ | 56.2 |  |  |

Note: F means forward, R means Reverse.

Table S3 Pearson correlation between soil chemical properties. Symbol “*” means signiﬁcant correlations (*P* < 0.05); “**” means novel signiﬁcant correlations (*P* < 0.001).

|  | pH | TC | TN | C/N | TP | TK | T Se | A Se |
| --- | --- | --- | --- | --- | --- | --- | --- | --- |
| pH | 1.000 | 0.243 | 0.308 | -0.247 | 0.169 | -0.444 | 0.384 | 0.608* |
| TC |  | 1.000 | 0.98** | 0.351 | 0.451 | -0.432 | 0.415 | 0.398 |
| TN |  |  | 1.000 | 0.167 | 0.445 | -0.432 | 0.486 | 0.471 |
| C/N |  |  |  | 1.000 | 0.116 | -0.166 | -0.151 | -0.212 |
| TP |  |  |  |  | 1.000 | -0.293 | -0.111 | -0.047 |
| TK |  |  |  |  |  | 1.000 | -0.291 | -0.248 |
| T Se |  |  |  |  |  |  | 1.000 | 0.903** |
| A Se |  |  |  |  |  |  |  | 1.000 |

Table S4 The detected concentration of antibiotic residues (μg kg^-1^).

| Sample | AMX | CA | TCN | DOX | OLF | ENR | ENY | KAN |
| --- | --- | --- | --- | --- | --- | --- | --- | --- |
| YTB2 | 3.44 | nd | nd | nd | nd | nd | nd | nd |
| CJB | 3.06 | nd | nd | nd | nd | nd | nd | nd |
| XQC | 0.49 | nd | nd | nd | nd | nd | nd | nd |
| SZP | 2.41 | nd | nd | nd | nd | nd | nd | nd |
| LZH | 0.65 | nd | nd | nd | nd | nd | nd | nd |
| SBY | 3.90 | nd | nd | nd | nd | nd | nd | nd |
| DDW | 0.00 | nd | nd | nd | nd | nd | nd | nd |
| SH | 0.00 | nd | nd | nd | nd | nd | nd | nd |
| LHB | 1.71 | nd | nd | nd | nd | nd | nd | nd |
| SKQ | 12.42 | nd | nd | nd | nd | nd | nd | nd |
| JYZ | 0.76 | nd | nd | nd | nd | nd | nd | nd |
| SQZ | 2.09 | nd | nd | nd | nd | nd | nd | nd |
| TZY2 | 3.44 | nd | nd | nd | nd | nd | nd | nd |
| MJW | 1.70 | nd | 0.45 | nd | nd | nd | nd | nd |
| TZY1 | 1.94 | nd | nd | nd | nd | nd | nd | nd |
| XJW | 0.00 | nd | nd | nd | nd | nd | nd | nd |
| HCT | 0.90 | nd | nd | nd | nd | nd | nd | nd |
| ZJW | 6.54 | nd | nd | nd | nd | nd | nd | nd |
| DLT | 0.00 | nd | nd | nd | nd | nd | nd | nd |
| WJW | 3.22 | nd | nd | nd | nd | nd | nd | nd |
| SDH | 5.09 | nd | nd | nd | nd | nd | nd | nd |
| YTB1 | 2.81 | nd | nd | nd | nd | nd | nd | nd |
| YTY | 3.30 | nd | 0.14 | nd | nd | nd | nd | nd |
| MJT | 0.00 | nd | nd | nd | nd | nd | nd | nd |

Notes: nd means not detected and the value was lower than the detection limit. AMX: amoxicillin; CA: clavulanic acid; TCN: tetracycline; DOX: doxycycline hydrochloride; OLF: ofloxacin; ENR: enrofloxacin; ENY: erythromycin; KAN: kalamycin.

Table S5 The absolute copy numbers of selenium resistant genes (copies g^-1^).

| Sample | ruvB | tehB | serD | sodB | recG | serC | srdB | mmtA | selA | selB | sodA | serA |
| --- | --- | --- | --- | --- | --- | --- | --- | --- | --- | --- | --- | --- |
| YTB2 | 1.09E+08 | 1.82E+06 | 6.72E+06 | 4.85E+05 | 5.80E+06 | 1.57E+05 | 5.58E+06 | 2.58E+05 | 3.14E+03 | 2.62E+05 | 1.04E+06 | 3.06E+05 |
| CJB | 1.41E+05 | 0.00E+00 | 5.54E+04 | 4.67E+04 | 2.04E+03 | 9.01E+01 | 2.93E+05 | 6.87E+04 | 0.00E+00 | 0.00E+00 | 1.51E+04 | 1.33E+02 |
| XQC | 2.03E+07 | 2.10E+05 | 9.03E+05 | 1.16E+05 | 8.15E+05 | 8.87E+03 | 1.23E+06 | 8.41E+04 | 2.96E+03 | 9.49E+04 | 1.68E+05 | 1.61E+04 |
| SZP | 1.85E+08 | 2.75E+06 | 9.22E+06 | 1.54E+06 | 8.94E+06 | 1.27E+05 | 6.64E+06 | 4.32E+05 | 8.20E+03 | 7.88E+05 | 1.06E+06 | 3.23E+05 |
| LZH | 1.81E+08 | 2.24E+06 | 1.25E+07 | 1.05E+06 | 7.20E+06 | 9.81E+04 | 3.44E+06 | 6.83E+05 | 1.97E+04 | 6.24E+05 | 3.49E+06 | 4.73E+05 |
| SBY | 3.35E+08 | 1.31E+06 | 7.64E+06 | 2.40E+05 | 5.81E+06 | 6.12E+04 | 7.12E+06 | 3.10E+05 | 1.18E+04 | 3.34E+05 | 4.52E+05 | 1.91E+05 |
| DDW | 2.04E+08 | 8.63E+06 | 1.84E+07 | 9.76E+05 | 1.35E+07 | 9.34E+04 | 4.67E+06 | 1.18E+06 | 2.48E+03 | 2.05E+06 | 7.58E+05 | 3.24E+05 |
| SH | 2.06E+08 | 8.00E+06 | 2.05E+07 | 6.49E+05 | 1.37E+07 | 1.43E+05 | 6.02E+06 | 9.94E+05 | 5.18E+03 | 1.62E+06 | 4.51E+05 | 6.18E+05 |
| LHB | 1.82E+08 | 1.17E+07 | 1.50E+07 | 2.01E+06 | 1.94E+07 | 1.25E+05 | 5.29E+06 | 1.05E+06 | 4.53E+03 | 1.70E+06 | 7.95E+05 | 2.57E+05 |
| SKQ | 1.93E+08 | 4.41E+06 | 1.41E+07 | 7.69E+05 | 2.62E+07 | 8.24E+04 | 5.49E+06 | 1.17E+06 | 2.29E+04 | 2.61E+06 | 4.26E+06 | 5.05E+05 |
| JYZ | 1.31E+08 | 1.24E+06 | 7.77E+06 | 3.84E+05 | 5.23E+06 | 5.57E+04 | 5.19E+06 | 6.20E+05 | 1.76E+04 | 1.21E+06 | 4.28E+06 | 2.85E+05 |
| SQZ | 1.38E+08 | 3.78E+06 | 1.90E+07 | 6.52E+05 | 6.12E+06 | 5.23E+04 | 4.21E+06 | 7.41E+05 | 4.60E+03 | 9.80E+05 | 2.63E+05 | 3.67E+05 |
| TZY2 | 4.16E+08 | 1.75E+06 | 2.02E+07 | 4.93E+05 | 1.27E+07 | 4.32E+04 | 3.15E+06 | 8.69E+05 | 4.95E+03 | 5.65E+05 | 5.52E+05 | 3.25E+05 |
| MJW | 4.54E+08 | 4.68E+06 | 1.66E+07 | 5.32E+05 | 1.34E+07 | 8.06E+04 | 6.22E+06 | 2.75E+06 | 1.58E+04 | 2.75E+06 | 2.80E+06 | 8.87E+05 |
| TZY1 | 5.40E+08 | 8.12E+06 | 2.70E+07 | 1.05E+06 | 3.05E+07 | 1.22E+05 | 7.41E+06 | 2.38E+06 | 1.12E+04 | 1.09E+06 | 2.45E+06 | 5.72E+05 |
| XJW | 2.06E+08 | 1.36E+06 | 1.88E+07 | 9.09E+05 | 8.17E+06 | 2.27E+05 | 1.59E+06 | 3.89E+05 | 5.49E+03 | 3.09E+05 | 4.77E+05 | 6.17E+05 |
| HCT | 1.08E+08 | 9.51E+05 | 5.65E+06 | 6.77E+05 | 5.10E+06 | 9.49E+05 | 1.63E+06 | 6.08E+05 | 9.20E+03 | 4.28E+05 | 4.75E+05 | 3.43E+05 |
| ZJW | 1.43E+08 | 1.89E+06 | 7.01E+06 | 1.09E+06 | 6.34E+06 | 6.59E+05 | 2.09E+06 | 1.15E+06 | 3.06E+03 | 5.39E+05 | 1.75E+06 | 3.43E+05 |
| DLT | 1.12E+08 | 1.27E+06 | 8.48E+06 | 2.55E+05 | 4.22E+06 | 6.47E+04 | 4.77E+05 | 4.49E+05 | 6.90E+03 | 6.83E+05 | 2.91E+06 | 9.93E+04 |
| WJW | 9.85E+07 | 5.75E+05 | 3.46E+06 | 1.24E+05 | 4.26E+06 | 3.91E+04 | 1.12E+06 | 4.04E+05 | 3.03E+03 | 6.30E+05 | 2.40E+06 | 6.66E+04 |
| SDH | 1.11E+08 | 4.55E+05 | 3.01E+06 | 1.58E+05 | 4.02E+06 | 5.30E+04 | 5.51E+05 | 6.67E+04 | 2.23E+03 | 6.01E+05 | 7.66E+04 | 5.17E+04 |
| YTB1 | 2.77E+08 | 1.39E+07 | 1.63E+07 | 7.88E+05 | 1.26E+07 | 2.27E+05 | 5.81E+06 | 1.23E+06 | 1.27E+04 | 1.33E+06 | 8.22E+05 | 6.61E+05 |
| YTY | 1.50E+08 | 4.34E+06 | 1.89E+07 | 1.09E+06 | 1.05E+07 | 4.66E+04 | 5.24E+06 | 5.63E+05 | 1.20E+04 | 6.11E+05 | 9.34E+05 | 2.52E+06 |
| MJT | 1.39E+08 | 6.82E+06 | 1.43E+07 | 7.79E+05 | 7.38E+06 | 4.13E+04 | 3.59E+06 | 1.06E+06 | 3.12E+03 | 7.61E+05 | 9.38E+05 | 4.26E+05 |
