# Supplementary material for: Natural selenium stress influences the changes of antibiotic resistome in seleniferous forest soils
Source: Environ Microbiome. 2022 May 15;17:26. doi: 10.1186/s40793-022-00419-z (PMC9107767; doi:10.1186/s40793-022-00419-z)
Supplement: Supplementary file 8 — Additional file 8: Text S1. Determination of total and available Se contents of soil. [file 40793_2022_419_MOESM8_ESM.docx]

**Determination of total selenium**

Total Se content was determined according to the method of Garcia et al [30] as follows: A 0.3500 g portion of dried soil samples were digested with 8 mL nitric acid and 2 mL perchloric acid for 12 hours at room temperature and then heated on an electrothermal heating plate at 200°C until the solution turned clear or colorless and accompanied by the release of white smoke. Subsequently, 15 mL of 5 M HCl was added to the solutions at 103oC for 1 min to reduce selenium (VI) to selenium (IV). After cooling to room temperature, 2.5 mL of 0.02 M EDTA was added to the solution in order to avoid interference from metal ions. The solution mixture was totally transferred to a 25 mL colorimetric tube and diluted to 25 mL with ultrapure water. After that, the solution mixture was analyzed using AFS-930 double channel atomic fluorescence spectrometry (Beijing Titan Instruments Co., Ltd, Beijing, China) [30]. Se content in the samples was determined by extrapolation to a standard curve of Se concentrations. The Se standard solution was obtained from the national standard substance center, Beijing, China.

The available Se content was measured using the following procedure: the soil sample was placed in a polypropylene centrifuge tube with the extractant 0.25 M KCl (V: m = 20:1 mL g-1); the mixture was mechanically shaken at 200 rpm for 2 h and then centrifuged at 4,000 rpm for 10 min at room temperature (25°C). After that, the supernatant was poured into a new polypropylene centrifuge tube and the residual solid phases were extracted with the same process once again. The supernatant obtained from the two steps was then mixed together. Subsequently, the following processes, including reduction, shielding and determination, were the same as those for the total Se content measurement.
